# Toll-like receptor 9 deficiency induces osteoclastic bone loss via gut microbiota-associated systemic chronic inflammation

**DOI:** 10.1038/s41413-022-00210-3

**Published:** 2022-05-27

**Authors:** Peng Ding, Qiyuan Tan, Zhanying Wei, Qiyu Chen, Chun Wang, Luyue Qi, Li Wen, Changqing Zhang, Chen Yao

**Affiliations:** 1 Department of Orthopedic Surgery, Shanghai Jiaotong University affiliated Sixth People’s Hospital, Shanghai, China; 2 Department of Endocrinology and Metabolism, Shanghai Jiaotong University affiliated Sixth People’s Hospital, Shanghai, China; 3 Department of Osteoporosis and Skeletal Disorders, Shanghai Jiaotong University affiliated Sixth People’s Hospital, Shanghai, China; 4 Department of Endocrinology and Metabolism, Second Affiliated Hospital of Soochow University, Suzhou, China; 5 Section of Endocrinology, Department of Internal Medicine, Yale University School of Medicine, New Haven, USA

**Keywords:** Bone, Pathogenesis

## Abstract

Toll-like receptors (TLRs) play pivotal roles in inflammation and provide important links between the immune and skeletal systems. Although the activation of TLRs may affect osteoclast differentiation and bone metabolism, whether and how TLRs are required for normal bone remodeling remains to be fully explored. In the current study, we show for the first time that TLR9^−/−^ mice exhibit a low bone mass and low-grade systemic chronic inflammation, which is characterized by the expansion of CD4^+^ T cells and increased levels of inflammatory cytokines, including TNF*α*, RANKL, and IL1*β*. The increased levels of these cytokines significantly promote osteoclastogenesis and induce bone loss. Importantly, TLR9 deletion alters the gut microbiota, and this dysbiosis is the basis of the systemic inflammation and bone loss observed in TLR9^−/−^ mice. Furthermore, through single-cell RNA sequencing, we identified myeloid-biased hematopoiesis in the bone marrow of TLR9^−/−^ mice and determined that the increase in myelopoiesis, likely caused by the adaptation of hematopoietic stem cells to systemic inflammation, also contributes to inflammation-induced osteoclastogenesis and subsequent bone loss in TLR9^−/−^ mice. Thus, our study provides novel evidence that TLR9 signaling connects the gut microbiota, immune system, and bone and is critical in maintaining the homeostasis of inflammation, hematopoiesis, and bone metabolism under normal conditions.

## Introduction

The close relationship between inflammation and bone metabolism has long been appreciated. Osteoclastic bone resorption stimulated by activated T cells and macrophages is the main cause of bone loss in inflammatory conditions such as RA and infectious diseases. Inflammatory cytokines, including TNF*α* and IL17 secreted by activated T cells and macrophages, work in concert to promote inflammation and osteoclastogenesis.^1^ Additionally, inflammation caused by sepsis leads to the inhibition of osteoblastic bone formation.^2^ Osteoclasts and immune cells are closely related. Osteoclasts are multinucleated cells formed by the fusion of monocyte–macrophage precursors and share many similarities with innate immune cells, including monocytes, macrophages, and dendritic cells, in terms of their origin and function. Since bone marrow is the primary site of hematopoiesis, harboring hematopoietic stem and progenitor cells (HSPCs) and mature immune cells (including B cells, macrophages and T cells), osteoclasts and immune cells share the same microenvironment and interact with each other in bone marrow. Osteoclasts have been shown to process, present and cross-present antigens, resulting in T cell activation. They also produce cytokines and immunomodulatory factors that affect immune responses.^3^ Reciprocally, activated T and B cells produce RANKL, which is one of the key signals driving osteoclast differentiation and activation. Moreover, OPG (the decoy receptor of RANKL) is highly expressed on B lymphocytes, and T cells are key regulators of OPG production in B cells and basal bone turnover.^4^ The imbalanced RANKL/OPG ratio caused by dysregulated T and B cell function contributes to the bone loss that is associated with HIV infection and induced by combination antiretroviral therapy.^5^ Furthermore, inflammatory cytokines, including TNF*α*, IL6 and IL1*β*, secreted by immune cells are potent proresorptive factors leading to bone loss in many inflammatory conditions, including rheumatoid arthritis (RA), inflammatory bowel disease (IBD), and HIV infection.^1,5,6^

In addition to classic inflammatory diseases such as RA, low-grade inflammation has been observed to play important roles in bone loss during aging and metabolic disorders.^7–9^ Studies have shown that inhibiting the production of the proinflammatory secretome by senescent cells prevents age-associated osteoporosis in mice.^8^ Inflammation related to T cell activation is an important cause of hyperparathyroidism- and estrogen deficiency-induced osteoporosis.^7,9^ Furthermore, increasing evidence suggests that alterations in the gut microbiota modulate inflammation and play a central role in estrogen deficiency-induced bone loss.^10,11^ The gut microbiome may affect bone physiology through different mechanisms; nevertheless, the modulation of the host immune system is thought to be an important pathway linking the gut microbiota and bone.^11^

Toll-like receptors (TLRs) play a pivotal role in innate immune responses against microbes through the recognition of pathogen-associated molecular patterns.^12^ TLR9 senses unmethylated CpG DNA from bacteria and viruses to initiate type I interferon (IFN) and proinflammatory cytokine production by immune cells.^13,14^ At present, the role of TLR9 in modulating inflammation is controversial. Previous studies have found that the activation of TLR9 by agonists induces or enhances inflammatory diseases such as experimental autoimmune encephalomyelitis, arthritis, vasculitis, myocarditis and periodonitis. The genetic ablation or molecular blockade of TLR9 alleviates or protects against diseases, supporting a disease-promoting role of TLR9 in these disorders.^15–21^ Stanbery et al.^22^ also showed that the dysregulated activation of TLR9 after genetic mutation led to fatal inflammatory disease driven by IFN-γ. Recently, however, increasing evidence has indicated that TLR9 suppresses inflammation in some disease models,^23–28^ and conflicting results have been reported based on different mouse models of SLE. Thus, further studies remain to be performed to determine whether TLR9 can restrain inflammation under normal conditions.

Most immune cells originate from bone marrow, suggesting a close interaction between bone and immunity. Indeed, in addition to their roles in mediating the immune response and inflammation, TLRs have been shown to regulate bone metabolism. TLRs link the immune and skeletal systems and are critical players in osteoimmunity.^29^ TLRs 1-9 are expressed in osteoclast progenitors (OCPs) and can have dual effects on osteoclastogenesis.^30^ The activation of TLRs can either increase osteoclast formation and bone resorption^31^ or arrest osteoclastogenesis.^32^ The role of TLR9 in osteoclasts is also controversial. TLR9 shows a downstream NF*κ*B signaling pathway similar to that of RANKL, but the activation of TLR9 only induces the formation of TRAP^+^ mononucleated cells, without the formation of mature osteoclasts.^33^ In vitro studies showed that the activation of TLR9 in bone marrow-derived macrophages by CpG-ODN suppressed RANKL-induced osteoclastogenesis, while CpG-ODN treatment promoted osteoclastogenesis in RANKL-primed osteoclast precursors.^34,35^ Interestingly, the pro-osteoclastogenic activity of TLR9 in committed OCPs is effected by inducing the production of TNF*α*, rather than as a direct effect of TLR9 signaling.^33^ Previous studies on the role of TLR9 in osteoclasts were mostly based on in vitro experiments involving activation by exogenous TLR9 ligands. However, little is known about the role of TLR9 in regulating bone remodeling in vivo under physiological conditions.

Here, using a TLR9-deficient (TLR9^−/−^) C57BL/6 mouse model, we showed for the first time that mice lacking TLR9 had a low bone mass due to increased osteoclastogenesis. Further mechanistic investigations revealed that TLR9^−/−^ mice exhibited low-grade systemic inflammation, which contributed to osteoclastic bone loss not only by the upregulation of proresorptive cytokines but also via myeloid-biased hematopoiesis. Moreover, we found that the increases in inflammation and bone loss were associated with alterations in the gut microbiota in TLR9^−/−^ mice. Thus, our results reveal a novel function of TLR9 in maintaining the homeostasis of inflammation and bone metabolism under physiological conditions.

## Results

### TLR9^−/−^ mice show low bone mass and increased osteoclastogenesis

**Fig. 1:**
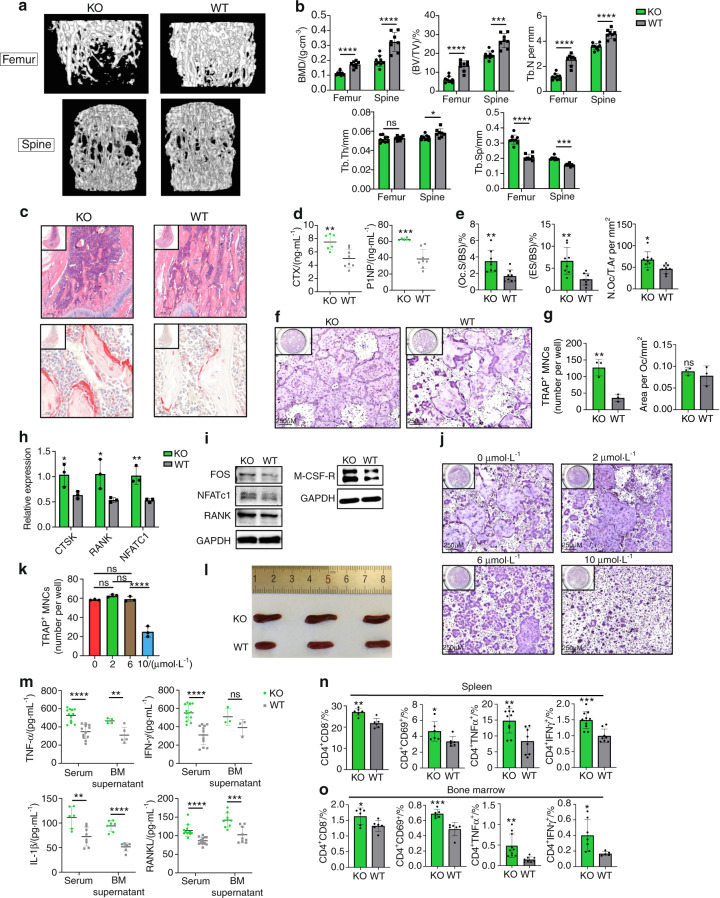
TLR9^−/−^ mice had low bone mass, increased osteoclastogenesis and systemic inflammation. **a** Representative 3D μCT images of femurs and L3 vertebrae from TLR9^−/−^ (KO) and wildtype (WT) mice. **b** Trabecular bone microarchitecture of femurs and L3 vertebrae showing bone mineral density (BMD), bone volume per tissue volume (BV/TV), number of trabeculae (Tb. N), trabecular thickness (Tb.Th) and trabecular separation (Tb.Sp). KO, *n* = 8; WT, *n* = 9. **c** Representative HE (upper panels) and TRAP (lower panels) stained images of distal femurs from each group. **d** Circulating CTX and P1NP levels. KO, *n* = 6; WT, *n* = 8. **e** Histomorphometric analysis of bone resorption indices. *n* = 8 per group. **f**–**h** In vitro osteoclastogenesis assay. **f** TRAP-stained osteoclast like cells (OCLs). **g** Quantification of number and area of TRAP^+^ multinucleated OCLs. **h** qPCR analysis of osteoclast signature genes’ expression at the end of assay. **i** Western blots showing expression of the indicated molecules in early osteoclast precursors. **j**–**k** In vitro osteoclastogenesis of wildtype BMNCs in the presence of TLR9 antagonist. **j** TRAP-stained OCLs. **k** Quantification of OCL numbers. **l** Spleens from 8-week-old male TLR9^−/−^ and wildtype mice. **m** Levels of TNF*α*, IFN*γ*, IL1*β*, and RANKL in serum and bone marrow supernatants. *n* = 3 to 13 per group. **n** Splenic *T* cell populations analyzed by flow cytometry. *n* = 6–10 per group. **o** The bone marrow *T* cell populations. *n* = 5–10 per group. The numbers in **n** and **o** represent the frequencies in total splenic or bone marrow cells. Eight-week-old sex-matched mice were used in **d**, **e** and **m**–**o**. One-way ANOVA with Turkey’s multiple comparison tests was conducted in **k**. In other panels, statistical significance was determined using an unpaired two-tailed t-test. Error bars represent the s.d. *P < 0.05, **P < 0.01, ***P < 0.001, ****P < 0.000 1 and ns *P* > 0.05

To investigate the role of TLR9 in osteoclastogenesis and bone metabolism, we first assessed the bone mass of TLR9^−/−^ (KO) and wild-type (WT, TLR9^+/+^) mice with a C57BL6/J genetic background^36,37^ by micro-CT analysis. Interestingly, we found that TLR9^−/−^ mice presented significantly lower trabecular bone mass in both the distal femur and vertebrae (Figs. 1a–c and S1a, b). There was no difference in cortical bone between the two groups (Fig. S1c–f). TLR9^−/−^ mice also showed elevated levels of bone resorption (CTX) and formation (P1NP) markers in their circulation, which indicates a high bone turnover status in TLR9^−/−^ mice (Fig. 1d). Furthermore, histomorphometric analysis showed significantly increased osteoclast numbers and higher bone resorption indices in TLR9^−/−^ mice (Fig. 1c, e). Although there was no significant difference in osteoblast numbers or surface area between the two groups, the mineral apposition rate (MAR) was higher in TLR9^−/−^ mice (Fig. S1g–j). Real-time PCR using calvarial osteoblasts from TLR9^−/−^ and WT mice showed that the expression of Alp and Osx was higher in the TLR9^−/−^ group, whereas there were no significant differences in other osteoblastic genes (Fig. S1k). The alkaline phosphatase (ALP) assay after in vitro osteoblast differentiation showed a slightly higher level of ALP in TLR9^−/−^ osteoblasts, but the level of statistical significance was not reached (Fig. S1l, m).

Next, we assessed the role of TLR9 in osteoclast differentiation by conducting an in vitro osteoclastogenesis assay. Primary nonadherent bone marrow mononuclear cells (BMNCs) from TLR9^−/−^ and WT mice were stimulated with RANKL and CSF1 for 5 days. We found that the number of osteoclast-like cells (OCLs) was substantially increased in the TLR9^−/−^ group, with no significant change in the area per OCL (Fig. 1f, g). Quantitative PCR results showed that TLR9^−/−^ OCLs (Day 5) presented higher expression of osteoclast signature genes, including CTSK, RANK and NFATc1, than WT OCLs (Fig. 1h). Western blot analysis also revealed that the protein levels of RANK, Fos, NFATc1 and CSF1R were upregulated in TLR9^−/−^ OCPs on Day 2 of in vitro culture (Fig. 1i). Furthermore, the TLR9^−/−^ OCPs showed a higher proliferation rate than the WT cells in response to CSF1 (Fig. S1n). These results indicate that TLR9^−/−^ bone marrow cells possess a greater potential to differentiate into osteoclasts than WT cells.

To verify whether the loss of osteoclast-intrinsic TLR9 was the main reason for the increase in osteoclastogenesis in TLR9^−/−^ mice, we cultured WT BMNCs in the presence of a TLR9-specific antagonist (ODN-2088, 0-10 μmol·L^−1^) to investigate whether the molecular blockade of TLR9 promotes osteoclastogenesis to a similar extent to the genetic ablation of TLR9. Our results showed that the TLR9 antagonist had no significant effect on osteoclast differentiation; however, we observed a suppressive effect on osteoclastogenesis when the TLR9 antagonist was applied at a very high concentration (10 μmol·L^−1^) (Fig. 1j, k). This result indicates that increased osteoclastogenesis in TLR9^−/−^ mice may not be caused by the loss of osteoclast-intrinsic TLR9 signaling. Alternatively, the increase in osteoclastogenesis in TLR9^−/−^ mice may be mediated by other mechanisms.

### TLR9^−/−^ mice exhibit chronic systemic inflammation

To further explore the mechanism underlying osteoclastic bone loss in TLR9^−/−^ mice, we first measured the levels of pro-osteoclastic cytokines in the circulation. Strikingly, the levels of inflammatory cytokines with potent osteoclastogenic activity, including TNF*α*, IL1*β* and RANKL, but not IL17 and IL6, were significantly elevated in serum samples from TLR9^−/−^ mice (Figs. 1m and S2a, b). In contrast, circulating OPG levels were decreased, which led to a significant increase in the RANKL/OPG ratio in TLR9^−/−^ mice (Fig. S2b). The circulating level of IFN*γ*, an important activator of both innate and adaptive immune responses, was also increased in TLR9^−/−^ mice (Fig. 1m). In line with the results obtained for circulating cytokines, higher TNF*α*, IL1*β* and RANKL levels and a lower OPG level were also found in the bone marrow supernatants of TLR9^−/−^ mice (Figs. 1m and S2b). In addition to higher levels of inflammatory cytokines in the circulation, TLR9^−/−^ mice exhibited enlarged spleens relative to their WT counterparts (Fig. 1l). These results suggest the presence of systemic inflammation in TLR9^−/−^ mice.

To investigate whether there were any changes in immune cells and which immune cells contributed to the increase in inflammatory cytokines seen in TLR9^−/−^ mice, we phenotyped immune cells from the bone marrow and spleen of TLR9^−/−^ and WT mice by flow cytometry. Our results showed that the TLR9^−/−^ mice presented increased proportions of splenic CD4^+^ and CD8^+^ T cells (Figs. 1n, o and S2c, d). We also observed significantly increases in bone marrow CD4^+^ T cells, with a trend of an increase in bone marrow CD8^+^ T cells in TLR9^−/−^ mice. Notably, TLR9^−/−^ mice showed more CD69^+^-activated CD4^+^ and CD8^+^ T cells than the WT mice in both their bone marrow and spleen (Figs. 1n, o and S2c, d). Intracellular cytokine analysis revealed that the numbers of TNF*α*- and IFN*γ*-producing CD4^+^ T cells were markedly increased in the bone marrow and spleen of TLR9^−/−^ mice (Fig. 1n, o). The TLR9^−/−^ mice presented more TNF*α*- and IFN*γ*-producing CD8^+^ T cells than the WT mice in the spleen but not in the bone marrow (Fig. S2c, d). There were also increased numbers of RANKL-producing splenic CD8^+^ T cells and TNF*α*-producing macrophages in the TLR9^−/−^ mice (Fig. S2c). In accordance with the flow cytometry results, TNF*α*, IFN*γ* and RANKL levels and the RANKL/OPG ratio were elevated in the culture supernatant of TLR9^−/−^ splenic CD4^+^ T cells (Fig. S2e). Thus, our findings indicate that CD4^+^ T cells are important contributors to the increased levels of inflammatory cytokines seen in TLR9^−/−^ mice.

B lymphocytes show a close relationship with bone cells, as the development and maturation of B cells take place in bone marrow. Therefore, we also investigated whether TLR9 deficiency affects B cells in bone marrow and in peripheral lymphoid tissue. Interestingly, we found that bone marrow and splenic B cell counts were decreased in the absence of TLR9 (Fig. S2c, d). B cells are RANKL and OPG producers, and mature B cells are the major source of OPG in bone marrow.^4^ To determine the levels of B cell-derived RANKL and OPG, we cultured purified splenic and bone marrow CD19^+^ B cells from TLR9^−/−^ and WT mice and measured secreted RANKL and OPG in the culture supernatant. Similar to CD4^+^ T cells, TLR9^−/−^ B cells showed increased production of soluble RANKL but a decreased level of OPG in the supernatant (Fig. S2f). As osteoblasts and bone marrow mesenchymal cells (BMSCs) are also important sources of RANKL and OPG, we further cultured osteoblasts and BMSCs from TLR9^−/−^ and WT mice. No differences in the levels of osteoblast- and BMSC-derived RANKL and OPG were found between the culture supernatants harvested from TLR9^−/−^ and WT cells (Fig. S2g). These results suggest that B cells also contribute to the imbalance in the RANKL/OPG ratio after the deletion of TLR9.

### Systemic inflammation is the major cause of increased osteoclastogenesis and bone loss in TLR9^−/−^ mice

**Fig. 2:**
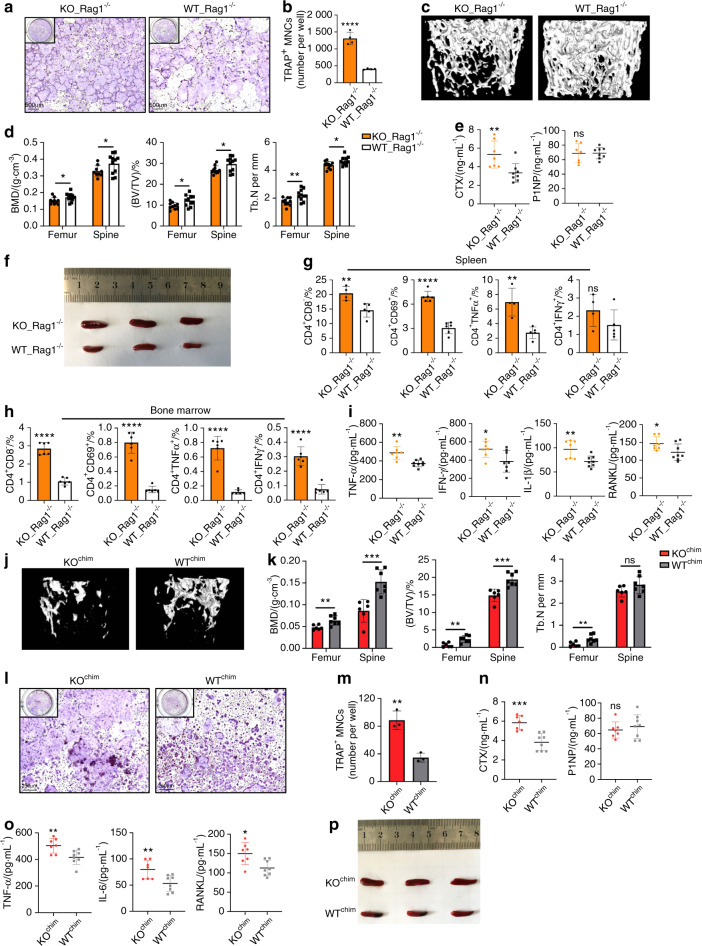
Systemic inflammation in TLR9^−/−^ mice plays an important role in osteoclastic bone loss. **a**, **b** Rag1^−/−^ BMNCs were cocultured with TLR9^−/−^ (KO) or wildtype (WT) splenocytes and stimulated with RANKL and CSF1 in a transwell system. The OCLs were stained by TRAP (**a**) and numbers quantified (**b**). **c**–**j** Adoptive transfer of TLR9^−/−^ splenocytes induced bone loss in Rag1^−/−^ mice. **c** Representative 3D μCT images of femurs from each group. **d** Trabecular BMD, BV/TV, and Tb. N of femurs and L3 vertebrae in recipient mice (KO_Rag1^−/−^, *n* = 10; WT_Rag1^−/−^, *n* = 11). **e** Serum CTX and P1NP. KO_Rag1^−/−^, *n* = 7; WT_Rag1^−/−^, *n* = 8. **f** Spleens of KO_Rag1^−/−^ and WT_Rag1^−/−^ mice. **g**–**h** Spleen (**g**) and bone marrow (**h**) T cell populations analyzed by flow cytometry. *n* = 4–6 mice per group. The numbers represent the frequencies in total splenic or bone marrow cells. **i** Serum cytokine levels. KO_Rag1^−/−^, *n* = 7; WT_Rag1^−/−^, *n* = 8. **j**–**p** The bone marrow transplantation experiment. **k** Trabecular BMD, BV/TV, and Tb. N of femurs and L3 vertebrae in the recipient mice (KO^chim^, *n* = 6; WT^chim^, *n* = 7). **l**–**m** In vitro osteoclastogenesis assay using BMNCs from KO^chim^ and WT^chim^ mice. **l** TRAP-stained OCLs. **m** Quantification of OCL numbers. **n** Circulating CTX and P1NP levels. KO^chim^, *n* = 7; WT^chim^, *n* = 8. **o** Serum TNF*α*, IL6 and RANKL levels. KO^chim^, *n* = 7; WT^chim^, *n* = 8. **p** Spleens of KO^chim^ and WT^chim^ mice. Error bars represent the s.d. *P *<* 0.05, **P < 0.01, ***P < 0.001, ****P < 0.000 1 and ns *P* > 0.05; statistical significance was determined using an unpaired two-tailed *t*-test

To investigate whether the inflammatory cytokines produced by immune cells were responsible for the increase in osteoclastogenesis in TLR9^−/−^ mice, we cultured splenocytes from TLR9^−/−^ and WT mice together with BMNCs from Rag1^−/−^ mice using a Transwell system. Rag1^−/−^ mice are devoid of T and B lymphocytes but show no change in bone mass relative to WT mice;^38^ thus, the use of an ideal source of BMNCs without T and B cell contamination and a Transwell culture system allows only soluble factors to flow freely. We found that the soluble factors produced by TLR9^−/−^ splenocytes induced a significantly higher count of osteoclasts from BMNCs than WT splenocytes (Fig. 2a, b). Furthermore, coculturing TLR9^−/−^ splenic CD4^+^ T cells, but not TLR9^−/−^ splenic B cells, with Rag1^−/−^ BMNCs significantly promoted osteoclast differentiation (Fig. S3a–d). This result further suggests that CD4^+^ T cells are the major contributors to increased osteoclastogenesis in TLR9^−/−^ mice. To confirm these in vitro results in vivo, we performed adoptive cell transfer experiments, in which splenocytes from TLR9^−/−^ or WT mice were adoptively transferred into Rag1^−/−^ recipient mice. Intriguingly, Rag1^−/−^ mice that received TLR9^−/−^ splenocytes (KO_Rag1^−/−^ mice) showed a lower bone mass than those that received WT splenocytes (WT_Rag1^−/−^ mice) (Figs. 2c, d and S3e). Accordingly, CTX level was also higher in KO_Rag1^−/−^ mice, but without any change in the P1NP level (Fig. 2e), indicating that the bone loss of KO_Rag1^−/−^ mice was due to increased bone resorption by osteoclasts.

Furthermore, the adoptive transfer of TLR9^−/−^ splenocytes recapitulated the inflammatory status in TLR9^−/−^ mice. KO_Rag1^−/−^ mice exhibited larger spleens than WT_Rag1^−/−^ mice (Fig. 2f) and showed increased counts of total CD4^+^ and CD8^+^ T cells as well as CD69^+^-activated CD4^+^ and CD8^+^ T cells in the bone marrow and spleen (Figs. 2g, h and S3f, g). Similar to TLR9^−/−^ mice, KO_Rag1^−/−^ mice exhibited elevated levels of TNF*α*, IFN*γ*, IL1*β*, RANKL and IL6 in their circulation (Figs. 2i and S3h). TNF*α*- and RANKL-producing CD4^+^ T-cell counts were markedly increased in the spleens of KO_Rag1^−/−^ mice (Figs. 2g and S3g). There were also more IFN*γ*-producing CD4^+^ and CD8^+^ T cells in the bone marrow of KO_Rag1^−/−^ mice (Figs. 2h and S3f). Consistent with the findings in TLR9^−/−^ mice, we found increased counts of total macrophages and TNF*α*-producing macrophages in the spleens of KO_Rag1^−/−^ mice (Fig. S3g). These observations further indicate that the splenocytes of TLR9^−/−^ mice are able to cause inflammation and bone loss in recipient mice. Together, these data show that the inflammation induced by immune cells plays an important role in mediating osteoclastic bone resorption in TLR9^−/−^ mice.

In addition to inflammation in the circulation and spleen, our data suggest that inflammation also occurred in the bone marrow of TLR9^−/−^ mice, since upregulated inflammatory cytokine levels in the bone marrow supernatant and an increased number of activated bone marrow T cells were observed. To verify the contribution of bone marrow inflammation and the hematopoietic compartment to bone loss in TLR9^−/−^ mice, we constructed bone marrow chimeras by transferring TLR9^−/−^ and WT bone marrow cells to 6-week-old lethally irradiated WT mice. Bone marrow chimerism was confirmed by the PCR genotyping of sex chromosomes in bone marrow cells (Fig. S3i). Six weeks after cell transfer, we found that mice that received TLR9^−/−^ bone marrow cells (KO^chim^) presented a significantly lower bone mass in the femur and spine than the mice that received WT bone marrow cells (WT^chim^) (Figs. 2j, k and S3j). KO^chim^ bone marrow cells showed an increased osteoclastogenic potential in vitro (Fig. 2l, m). Accordingly, KO^chim^ mice presented a significantly elevated level of CTX, whereas the level of P1NP was similar between the two groups (Fig. 2n). These results indicate that osteoclastic bone resorption is the major cause of bone loss in KO^chim^ mice. Furthermore, similar to TLR9^−/−^ mice, KO^chim^ mice also exhibited enlarged spleens, increased circulating TNF*α*, IL6 and RANKL levels and decreased circulating OPG levels (Figs. 2o, p and S1k). A trend toward higher circulating IL1*β* and IFN*γ* levels was also observed in TLR9^−/−^ mice (Fig. S3k). These results demonstrate that the transplantation of TLR9^−/−^ bone marrow cells promoted bone loss and a low level of systemic inflammation in recipient mice, further indicating that bone marrow inflammation and hematopoietic cells play an important role in osteoclastic bone loss in TLR9^−/−^ mice.

**Fig. 3:**
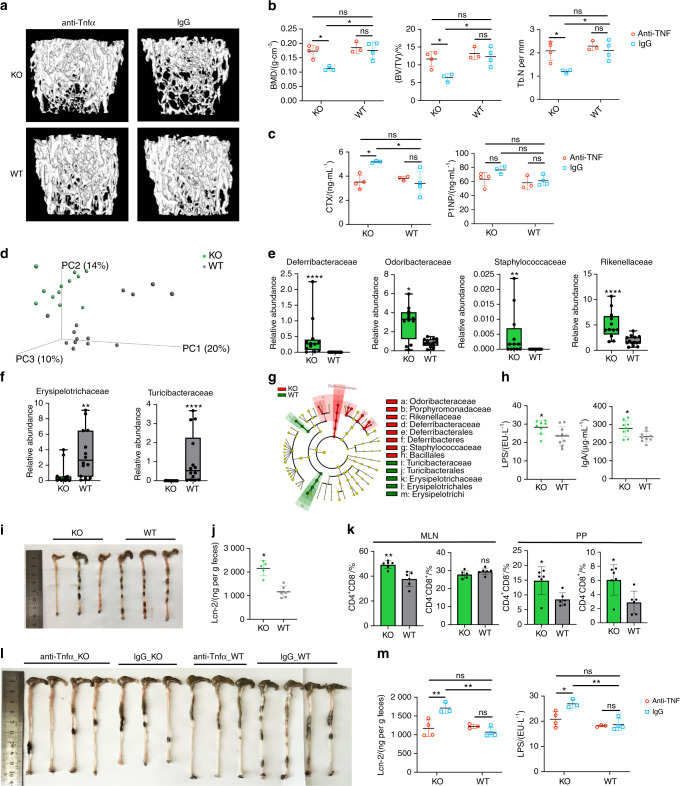
The anti-TNF*α* therapy experiment and the altered gut microbiota composition and intestinal inflammation in TLR9^−/−^ mice. **a**–**c**, **l**, **m** The anti-TNF*α* therapy experiment. In all panels, *n* = 3 in IgG_KO and anti-TNF*α*_WT group; *n* = 4 in anti-TNF*α*_KO and IgG_WT group. **a** Representative 3D μCT reconstructions of femurs from each group. **b** Trabecular BMD, BV/TV, and Tb. N of femurs in each group. **c** Serum CTX and P1NP levels. **d** 3D PCoA of 16S rRNA sequencing of fecal microbiota from 8-week sex-matched TLR9^−/−^ (KO) and wildtype (WT) mice. Each dot represents a fecal microbiota from one mouse. KO, *n* = 12; WT, *n* = 14. **e**–**f** Relative abundance of the significantly upregulated families (*P* < 0.05) in KO (**e**) and WT (**f**) group. Boxplots represent median and quantiles. **g** Cladogram of Linear discriminant analysis Effect Size (LEfSe) analysis showing the differentially abundant bacteria between KO and WT groups. **h** Circulating LPS and IgA levels. *n* = 8–10 per group. **i** Large intestines of 8-week-old male KO and WT mice. **j** Fecal Lcn-2 levels. KO, *n* = 5; WT, *n* = 6. **k** Flow cytometry analysis of T cell populations in MLN and PP. *n* = 6 per group. The numbers represent the frequencies in total MLN or PP cells. **l**, **m** Ameliorated gut inflammation after anti-TNF*α* therapy. **l** Large intestines from all treated mice. **m** Serum LPS and fecal Lcn-2 levels. Mann–Whitney *t* test was used in **e** and **f**. Two-way ANOVA with multiple comparisons (Turkey’s test) were used in **b, c** and **m**. An unpaired two-tailed t-test was applied in other panels. Error bars represent the s.d. *P < 0.05, **P < 0.01, ****P < 0.000 1 and ns *P* > 0.05

The secretion of proinflammatory cytokines is one of the basic elements of inflammation,^39^ and one key feature of the systemic inflammation observed in TLR9^−/−^ mice is increased levels of multiple pro-osteoclastogenic inflammatory cytokines. Among these cytokines, TNF*α* is a central player in inflammatory bone loss,^1^ and TLR9^−/−^ mice showed significantly increased TNF*α* levels in the circulation and bone marrow. To determine the role of TNF*α* in bone loss in TLR9^−/−^ mice, we treated WT and TLR9^−/−^ mice with an anti-TNF*α* antibody or control IgG. In line with previous reports showing that the blockade of TNF*α* decreased bone resorption,^40^ we found that the trabecular bone volume of TLR9^−/−^ mice was significantly increased after anti-TNF*α* treatment and became similar to the bone volume of IgG-treated WT mice (TNF*α*_KO versus IgG_WT) (Figs. 3a, b and S4a). Anti-TNF*α* therapy also resulted in a significantly decreased CTX level, with no significant change in the P1NP level in TLR9^−/−^ mice (Fig. 3c). Interestingly, circulating IL6 and TNF*α* levels in TLR9^−/−^ mice were reduced after anti-TNF*α* therapy (Fig. S4b). We also observed trends toward lower circulating RANKL and IFN*γ* levels in TLR9^−/−^ mice after anti-TNF*α* therapy (Fig. S4b). Furthermore, anti-TNF*α* therapy decreased the frequency of splenic CD4^+^ and CD8^+^ T cells in TLR9^−/−^ mice (Fig. S4c). The restoration of bone mass by anti-TNF*α* therapy suggests an important role of TNF*α* in the bone loss of TLR9^−/−^ mice. Taken together, the above results indicate that the chronic systemic inflammation observed in TLR9^−/−^ mice is the major cause of increased osteoclastogenesis and subsequent bone loss.

### TLR9 deficiency alters the gut microbiota composition and induces intestinal inflammation

Although our data demonstrated chronic inflammation resulting in bone loss in TLR9^−/−^ mice, the source of this inflammation was not clear. TLR signaling has been shown to affect gut microbial ecology, and an altered gut microbial composition is an important driving force in inflammation and diseases.^41,42^ Recent studies have further suggested that the gut microbiota plays a key role in inflammatory bone loss.^7,43^ We therefore envision that TLR9 deficiency may alter the gut microbial composition, which could be the primary source of inflammation.

To test our hypothesis, we first performed 16S rRNA sequencing of the microbiome using feces from WT and TLR9^−/−^ mice. Principal coordinate analysis (PCoA) of the microbiomes of these mice showed segregation between the two groups (Fig. 3d). Both the richness of the gut flora and Shannon’s diversity were similar between WT and TLR9^−/−^ mice (Fig. S4e). Relative abundance at the family level showed differences in the composition of the microbiota between the two groups (Fig. S4f). We observed a TLR9^−/−^ microbiota signature associated with an increase in the families Deferribacteraceae, Odoribacteraceae, Rikenellaceae and Staphylococcaceae and a decrease in the families Erysipelotrichaceae and Turicibacteraceae (Fig. 3e, f). Notably, Deferribacteraceae, Odoribacteraceae and Rikenellaceae, which are gram-negative bacteria, presented higher abundance in TLR9^−/−^ mice than in WT mice. This observation was further supported by the linear discriminant analysis (LDA) effect size (LEfSe) results (Figs. 3g and S4g). Interestingly, the phylum Deferribacteres was specifically enriched in the TLR9^−/−^ group (Figs. 3g). An analysis of the ten most abundant species showed that two gram-negative bacteria, *Mucispirillum schaedleri* (*M. schaedleri*, of the phylum Deferribacteres) and *Parabacteroides distasonis* (*P. distasonis*, of the phylum Bacteroidetes), presented significant increases in TLR9^−/−^ mice (Fig. S4h). Interestingly, both *M. schaedleri* and *P. distasonis* have been reported to be associated with IBDs.^44,45^ Taken together, our results showed that the gut microbiota was altered such that pathologic properties were increased in the absence of TLR9.

Next, we investigated whether the altered gut microbiota in TLR9^−/−^ mice affected systemic immunity. To this end, we tested the circulating levels of IgA and lipopolysaccharide (LPS), an endotoxin produced mainly by gram-negative bacteria. Interestingly, both circulating LPS and IgA levels were higher in TLR9^−/−^ mice (Fig. 3h). The increased LPS level was in accordance with the higher abundance of gram-negative bacteria in TLR9^−/−^ mice. We also assessed the length of the large intestine from the ileocaecal junction to the anus in the mice. Intriguingly, the large intestine was significantly shorter in TLR9^−/−^ mice than in their WT counterparts (Fig. 3i), which indicates intestinal inflammation^46^ in TLR9^−/−^ mice. We then measured fecal Lipocalin 2 (Lcn-2), a marker of colitis, and found that TLR9^−/−^ mice presented higher levels of fecal Lcn-2 than WT mice (Fig. 3j). Next, we analyzed immune cells in mucosal-associated lymphoid tissues, and a higher proportion of CD4^+^ T cells was observed in the mesenteric lymph nodes (MLNs) and Peyer’s patches (PPs) of TLR9^−/−^ mice (Fig. 3k). TLR9^−/−^ mice also showed increased CD8^+^ T cell counts in their PPs (Fig. 3k). Furthermore, after treatment with anti-TNF*α*, the length of the large intestine of the TLR9^−/−^ mice was increased to the same length observed in the WT mice (Fig. 3l). Anti-TNF*α* treatment also significantly reduced the levels of fecal Lcn-2 and circulating LPS (Fig. 3m) in TLR9^−/−^ mice. CD4^+^ and CD8^+^ T cell counts were significantly decreased in the PP of TLR9^−/−^ mice after treatment with anti-TNF*α* (Fig. S4d). Together, these results suggest the presence of subclinical chronic intestinal inflammation in TLR9^−/−^ mice.

### An altered gut microbiota plays an important role in systemic inflammation and subsequent bone loss in TLR9^−/−^ mice

**Fig. 4:**
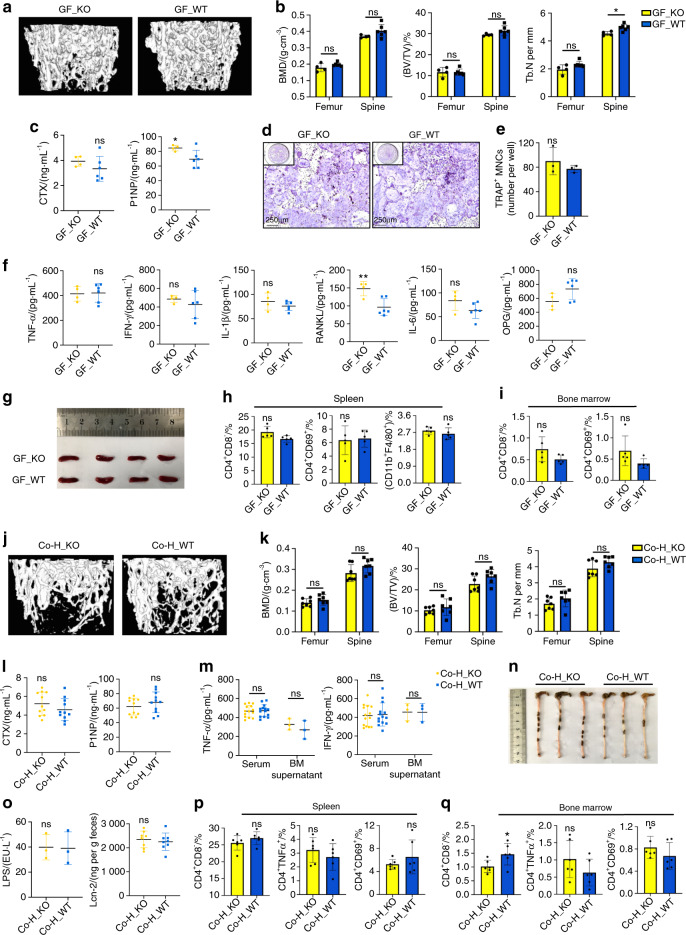
Altered gut microbiota played an important role in the systemic inflammation and bone loss in TLR9^−/−^ mice. **a**–**i** Phenotypic study of 8-week-old male germ-free (GF) TLR9^−/−^ and wildtype mice. **a** Representative 3D μCT images of the femurs. **b** Trabecular BMD, BV/TV, and Tb. N of femurs and L3 vertebrae in each group. GF_KO, *n* = 4; GF_WT, *n* = 6. **c** Circulating CTX and P1NP levels. GF_KO, *n* = 4; GF_WT, *n* = 6. **d**–**e** In vitro osteoclastogenesis using BMNCs from the GF mice. **d** TRAP-stained OCLs. **e** Quantification of OCL numbers. **f** Circulating cytokine levels. GF_KO, *n* = 4; GF_WT, *n* = 6. **g** Spleens from each group. **h**–**i** Flow cytometry analysis of splenic CD4^+^ T cells and macrophages (**h**) and bone marrow CD4^+^ T cells (**i**). *n* = 5 per group. **j**–**r** The cohousing experiment. **j** Representative 3D μCT images of the femurs. **k** Trabecular BMD, BV/TV, and Tb. N of femurs and L3 vertebrae in male Co-H_KO and Co-H_WT mice. *n* = 7 per group. **l** Serum CTX and P1NP levels. *n* = 11 per group. **m** TNF*α* and IFN*γ* levels. Serum, *n* = 14 per group; BM supernatant, *n* = 3 per group. **n** Large intestines from male Co-H_KO and Co-H_WT mice. **o** Serum LPS and fecal Lcn-2 levels. LPS, *n* = 3 per group; Lcn-2, *n* = 8 per group. **p**, **q** Spleen (**p**) and bone marrow (**q**) CD4^+^ T cell populations by flow cytometry. *n* = 6 per group. Twelve-week-old sex-matched mice were used in **l**, **m** and **o**–**q**. The numbers in **h**, **i**, **p** and **q** represent the frequencies in total splenic or bone marrow cells. Significance was determined using an unpaired two-tailed *t*-test. Error bars represent the s.d. *P *<* 0.05, **P < 0.01 and ns *P* > 0.05

To verify whether the altered gut microbiota contributes to the inflammation and changes in bone mass observed in the absence of TLR9, germ-free (GF) TLR9^−/−^ and WT mice were rederived via hysterectomy and maintained in gnotobiotic facilities for 8 weeks. Consistent with our hypothesis, the trabecular bone mass of GF TLR9^−/−^ mice was restored to the same level observed in their WT counterparts at the age of 8 weeks (Figs. 4a, b and S5a). Although the P1NP level was still higher in GF TLR9^−/−^ mice, the CTX level of GF TLR9^−/−^ mice was similar to that of GF WT mice (Fig. 4c) and was markedly lower than that of conventional TLR9^−/−^ mice (Fig. 1d). An in vitro osteoclastogenesis assay using BMNCs from GF TLR9^−/−^ and WT mice also showed a similar number of OCLs between the two groups (Fig. 4d, e).

Then, we compared the inflammatory status of GF TLR9^−/−^ and WT mice. With the exception of a higher level of circulating RANKL in GF TLR9^−/−^ mice, no significant differences in the levels of other cytokines were observed between the two groups (Fig. 4f). Notably, the circulating levels of TNF*α*, IL1*β* and IFN*γ* were markedly decreased in GF TLR9^−/−^ mice compared to TLR9^−/−^ mice raised under conventional conditions (Figs. 4f and 1m). Furthermore, the size of the spleen was reduced in GF TLR9^−/−^ mice and was similar to that observed in GF or conventional WT mice (Figs. 1l and 4g). No significant difference in the proportions of splenic CD4^+^ and CD8^+^ T cells, macrophages or CD69^+^-activated T cells was found between the two GF groups (Figs. 4h and S5b). Although there was a slight increase in bone marrow CD8^+^ T cells in GF TLR9^−/−^ mice, the frequencies of other bone marrow T cell populations were similar between the two groups (Figs. 4i and S5b). Although GF TLR9^−/−^ mice presented fewer splenic and bone marrow B cells (Fig. S5b), the difference in the proportion of bone marrow B cells between GF TLR9^−/−^ and WT mice was significantly decreased compared to the difference between conventional TLR9^−/−^ and WT mice (Fig. S2d). Together, these results showed decreased bone resorption along with lower inflammation levels in GF TLR9^−/−^ mice, further suggesting that the gut microbiota is an important source of inflammation in TLR9^−/−^ mice.

Cohousing can facilitate microbial transfer among individual animals.^47^ To gain further support for findings obtained in GF mice, we cohoused newly weaned WT and TLR9^−/−^ mice for 9 weeks. By sequencing the gut microbiota from fecal samples of cohoused mice, we found that the differences in the gut microbiota between the two genotypes were normalized after cohousing (Fig. S5c–h). The abundances of the signature TLR9^−/−^ families were similar between the two cohoused groups, with an increase in Deferribacteraceae in the WT mice and a decrease in Odoribacteraceae and Rikenellaceae in the TLR9^−/−^ mice after cohousing (Fig. S5f). At the species level, the abundances of both *M. schaedleri* and *P. distasonis* were similar between the two cohoused groups (Fig. S5h). Thus, our results confirmed that cohousing TLR9^−/−^ and WT mice facilitated microbial transfer and normalized the difference in the gut microbiota between the two groups.

Next, by investigating the effect of cohousing on the bone phenotype, we discovered that the trabecular bone density of TLR9^−/−^ and WT mice became similar after cohousing (Figs. 4j, k and S5i–k). The circulating CTX level in cohoused TLR9^−/−^ mice was decreased to a level similar to that in the cohoused WT mice, and no difference in P1NP levels was found between these two groups (Fig. 4l). Cohousing with WT mice also lowered TNF*α* and IFN*γ* levels in the circulation and bone marrow of TLR9^−/−^ mice, and no difference in the levels of circulating and bone marrow TNF*α*, IL6, IFN*γ* and OPG was found between the cohoused TLR9^−/−^ and WT mice (Figs. 4m and S5l). Although RANKL and IL1*β* levels were still higher in the cohoused TLR9^−/−^ mice than in their WT counterparts (Fig. S5l), their levels in the bone marrow of cohoused TLR9^−/−^ mice were significantly lower than those in the bone marrow of single-housed TLR9^−/−^ mice (Fig. 1m). Interestingly, cohousing also normalized spleen size and large intestine length between the TLR9^−/−^ and WT mice (Figs. 4n and S5m). Fecal Lcn-2 and serum LPS levels were similar between the two cohoused groups (Fig. 4o). No significant differences in the T cell populations of the MLNs, PPs, spleen and bone marrow were observed between TLR9^−/−^ mice and WT mice after cohousing (Figs. 4p–q and S5n–p). Taken together, the above results are in line with the findings in GF mice, further suggesting that the alteration of the gut microbiota is critical for the development of inflammation and subsequent bone loss in TLR9^−/−^ mice.

### Myeloid-biased hematopoiesis plays an important role in inflammatory bone loss in TLR9^−/−^ mice

Although our data showed that immune cell-produced inflammatory cytokines promoted osteoclastogenesis in TLR9^−/−^ mice in vivo, they did not fully explain why TLR9^−/−^ BMNCs generated more osteoclasts than WT cells in vitro after induction with the same concentrations of osteoclastogenic cytokines. Recently, emerging evidence has shown that chronic inflammation disturbs the normal homeostasis of hematopoiesis and results in myeloid skewing.^48,49^ Since TLR9^−/−^ mice showed increased inflammatory cytokines and fewer B lymphocytes in their bone marrow, we hypothesize that the chronic inflammation observed in TLR9^−/−^ mice may result in myeloid-biased hematopoiesis, which may further promote osteoclastogenesis by increasing the frequency of OCPs in the bone marrow.

**Fig. 5:**
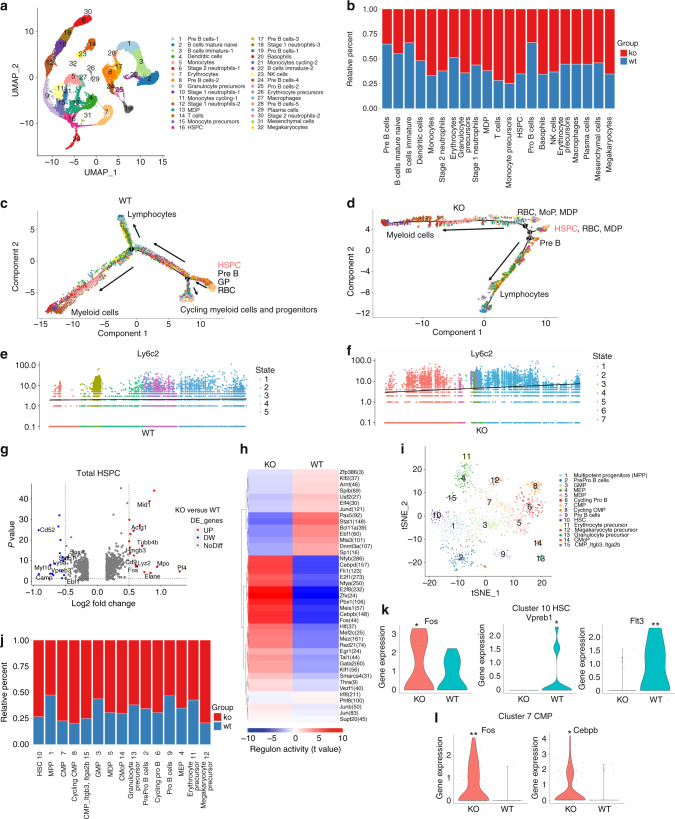
Single cell RNA sequencing reveals myeloid-biased hematopoiesis in the bone marrow of TLR9^−/−^ mice. **a** scRNA-seq of BMNCs from TLR9^−/−^ (KO) and wildtype (WT) mice. UMAP clustering of 36263 single-cell transcriptomes colored by significant cell-type clusters. **b** Proportions of KO and WT cells in each cell type. Clusters of the same cell types were combined for analysis. **c**, **d** Monocle trajectories of WT (**c**) and KO (**d**) cells colored by cluster as in **a**. Expression of monocyte marker Ly6c2 along pseudotime (from left to right) within the WT (**e**) and KO (**f**) bone marrow cells. **g**–**l** Mapping of HSPCs. **g** The differentially expressed genes (DEGs) between KO and WT cells within total HSPCs (DEGs with Log_2_[fold change] > 0.5 and *P* value < 0.05 were considered significant). Red dots, upregulated genes in TLR9^−/−^ cells; blue dots, downregulated genes in TLR9^−/−^ cells. **h** SCENIC analysis revealed differences of specific regulons activities between KO and WT cells in the entire HSPCs. **i** The reclustered HSPCs (cluster 16 in **a**). **j** Proportions of KO and WT cells in each subcluster. X axis represents cluster number and cell type. **k** Differential expressions of Fos, Vpreb1 and Flt3 in HSC (cluster 10 in **i**). **l** Expressions of Cebpb and Fos in CMP (cluster 7 in **i**). Statistical significance was determined using Wilcoxon test; *P *<* 0.05 and **P < 0.01

Bone marrow is a highly heterogeneous tissue comprised of many cell types. To further dissect the effect of TLR9 on the development of different cell lineages, we employed single-cell RNA sequencing (scRNA-seq) to examine differentially expressed genes (DEGs) in BMNCs from TLR9^−/−^ and WT mice using the 10x Genomics Chromium platform. Following rigorous quality control, we compiled gene expression data for clustering analyses from 36 263 cells (18 664 and 17 599 cells from TLR9^−/−^ and WT mice, respectively). This revealed 32 distinct populations visualized as uniform manifold approximation and projection (UMAP) embeddings. Population nomenclature was based on specific gene expression, and we identified 10 clusters of B cells, 5 clusters of macrophages/monocytes and precursors, 5 clusters of neutrophils, 2 clusters of erythrocytes and precursors, and 1 cluster of each of the following cell types: T cells, dendritic cells, NK cells, monocyte-dendritic cell precursors (MDPs), HSPCs, mesenchymal cells, basophils, plasma cells and megakaryocytes (Figs. 5a and S6a). Consistent with our earlier results, TLR9^−/−^ mice (KO) presented fewer B cells than WT mice; however, the scRNA-seq results revealed an increase in myeloid cell lineages, including monocytes, neutrophils and their progenitors. Greater numbers of T cells and HSPCs were also observed in TLR9^−/−^ mice (Figs. 5b and S6b, c).

To further analyze the differences in the development of bone marrow cells between the two groups, different clusters of cells were ordered along a trajectory, and distinct differentiation trajectories were identified in the TLR9^−/−^ (KO) and wild-type (WT) cells (Fig. 5c, d). Before definitive lineage commitments (Branch 1), the WT cells developed into a cluster of cycling myeloid cells and progenitors (Branch 2) (Figs. 5c and S7a, b). Interestingly, we found that the majority of WT Cluster 11 and 21 monocytes were within branch 2 (Fig. S8a, right panel), indicating that a large portion of WT monocytes (comprising Clusters 5, 11 and 21 in Fig. 5a) were going through the cell cycle and were not in a state of differentiation. In contrast to WT cells, 3 branches of KO cells were identified (Figs. 5d, S7a, c and S8a), and there was a markedly shorter path before lineage commitment (KO, from branch 3 to branch 1 or 2 in Fig. 5d, versus WT, from branch 2 to 1 in Fig. 5c). Clusters 11 and 21 of monocytes from the KO group were mainly on a differentiation trajectory rather than a cycling state (Fig. S8a, left panel). Pseudotime gene analysis also showed that monocyte signature gene expression increased more rapidly over time in TLR9^−/−^ bone marrow cells (Fig. 5e, f and S8b–d). Taken together, these results suggest the occurrence of faster lineage commitment and more rapid monocyte differentiation in TLR9^−/−^ bone marrow cells.

Next, we sought to obtain insight into the cluster of HSPCs (Cluster 16), which was enriched for markers of stem cells (CD34, Ifitm1, and Hlf).^50^ The analysis of DEGs showed that TLR9^−/−^ HSPCs exhibited significantly increased expression levels of myeloid signature genes (Lyz2, Mpo, Fos, Elane, CD9),^51,52^ whereas lymphoid differentiation genes (Sox4, Satb1, Vpreb3 and Myl10)^53–55^ were downregulated in TLR9^−/−^ HSPCs (Fig. 5g). We then performed a single-cell regulatory network inference and clustering (SCENIC) analysis to investigate the difference in the activity of transcription factors (TFs) between WT and TLR9^−/−^ HSPCs. Accordingly, we found increased activities of myeloid and stem cell-related TFs (Cebpb, Fos, Jun, Egr1 and Hlf) and decreased activities of B cell development-related TFs (Pax5, Ebf1 and Klf2) in TLR9^−/−^ HSPCs (Fig. 5h). These findings further indicated a bias toward myelopoiesis in TLR9^−/−^ HSPCs. Interestingly, TLR9^−/−^ HSPCs showed significantly higher activity of Cebpd (157 predicted downstream genes), an acute-phase inflammatory response gene,^56,57^ suggesting the priming of TLR9^−/−^ HSPCs by inflammatory signals.

Due to the heterogeneity of HSPCs, these cells were further divided into 15 subclusters based on specific gene expression. The subclusters included HSCs (Hlf, Ifitm1 and low expression of CD34), MPP cells (CD34 and moderate expression of Hlf and Ifitm1), 2 clusters of CMPs (Kit and low expression of Flt3 and Ly6a), clusters of myeloid progenitors including GMP (Mpo and Lyz2), MDP (Mpo and Irf8), cMoP (Lyz2 and Ly6c2) and granulocyte precursor cells (Lyz2 and Chil3), clusters of B cell progenitors (enriched for Flt3, Ebf1, Dntt, CD79a and CD79b), MEP cells (Gata2 and Car1), erythrocyte progenitors (high expression of Car1) and megakaryocyte progenitors (Pbx1, Pf4 and Vwf) (Figs. 5i and S8e). TLR9^−/−^ cells (KO) predominated in each subcluster of HSPCs, especially in HSCs, CMPs, MDPs and cMoPs (Fig. 5j). Within HSCs (Cluster 10), TLR9^−/−^ cells showed significantly higher expression of Fos and lower expression of Flt3 and Vpreb1 (Fig. 5k), indicating a trend toward myeloid lineage commitment in TLR9^−/−^ HSCs. Similarly, the TLR9^−/−^ group presented higher expression of Cebpb and Fos in CMPs (Cluster 7) (Fig. 5l). Higher expression of myelopoietic genes (Mpo, Cebpb, Elane, Ms4a3 and Fos) and downregulation of lymphopoietic genes (Sox4, Myl10, Pax5 and Ly6d) were also observed in other subclusters of interest (Fig. S8f, g). Taken together, these results suggest the occurrence of myeloid-biased hematopoiesis in TLR9^−/−^ bone marrow in early HSPCs.

**Fig. 6:**
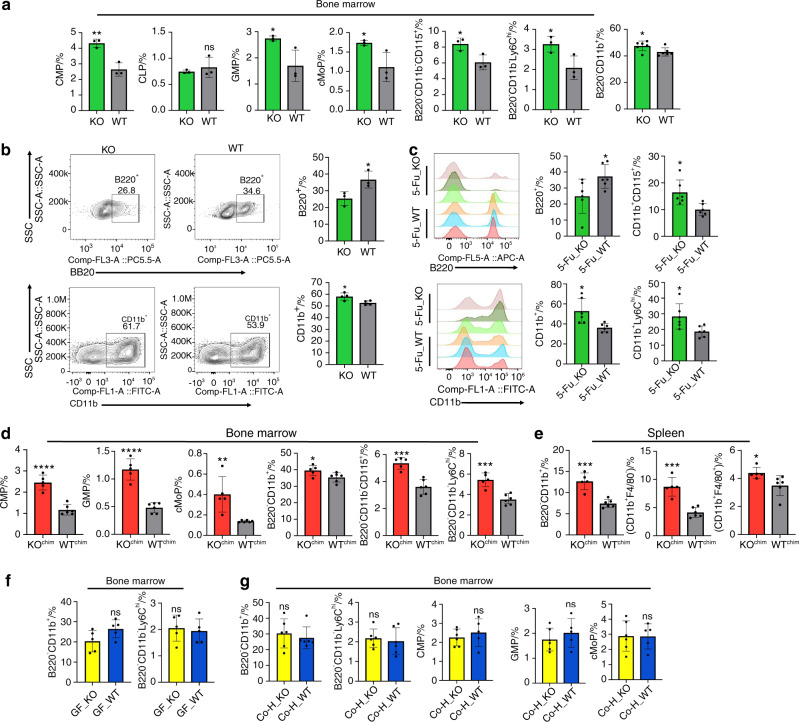
Myeloid-biased hematopoiesis plays an important role in the inflammatory bone loss in TLR9^−/−^ mice. **a** Proportion of bone marrow progenitor cells, myeloid cells and osteoclast progenitors in TLR9^−/−^ and wildtype mice analyzed by flow cytometry. *n* = 3 or 6 per group. The data of B220^-^CD11b^+^ cells was pool of results from two independent experiments. In other panels, representative data from one of two independent experiments were shown. **b** In vitro differentiation of hematopoietic stem cells. LSK^+^cells were sorted by FACS and cultured in liquid medium under lymphoid (upper panels) or myeloid (lower panles) supportive conditions for 12 or 7 days, respectively. At the end of assay, cells were stained with antibodies to B220 or CD11b to quantify the output of lymphoid or myeloid cells by flow cytometry. **c** Eight-week-old male TLR9^−/−^ (KO) and wildtype (WT) mice were exposed to the chemotherapy drug 5-FU and populations of bone marrow myeloid cells and B220^+^ cells were analyzed by flow cytometry 12 days after treatment. Left 2 panels, representative histograms showing populations of B220^+^ cells and CD11b^+^ cells after exposure to 5-FU. Right 4 panels, proportions of myeloid and B220^+^ cells in bone marrow of KO and WT mice after 5-FU treatment. *n* = 6 per group in all panels. Flow cytometry analysis of bone marrow (**d**) and splenic (**e**) myeloid cells in the bone marrow chimeric mice models. *n* = 5 and 6 in KO^chim^ and WT^chim^ group, respectively. **f** Flow cytometry analysis of bone marrow myeloid cells in the germ-free (GF) mice models. *n* = 5 per group in all panels. **g** Flow cytometry analysis of bone marrow myeloid cells in the 12-week-old male and female (sex-matched) cohoused mice. *n* = 6 in Co–H_KO and Co–H_WT group in all panels. The numbers in all flow cytometry results represent the frequencies in total splenocytes, bone marrow cells or cultured cells. Error bars represent the s.d. *P < 0.05, **P *<* 0.01, ***P < 0.001, ****P < 0.000 1 and ns *P* > 0.05; statistical significance was determined using an unpaired two-tailed *t*-test

Next, we performed flow cytometry analysis to corroborate the results obtained from scRNA-seq. We found that there were more myeloid progenitor cells, including GMP, CMP and cMoP cells, in the bone marrow of TLR9^−/−^ mice (Fig. 6a). The proportion of common lymphoid progenitors in the bone marrow was similar between the two groups (Fig. 6a). Additionally, the number of total CD11b^+^ myeloid cells was increased in the bone marrow of TLR9^−/−^ mice (Fig. 6a). Interestingly, we also identified increased counts of osteoclast precursor cells (B220^-^CD11b^-^CD115^+^ and B220^−^CD11b^−^Ly6c^hi^ cells) in the bone marrow of TLR9^−/−^ mice (Fig. 6a).

To expand our study on hematopoiesis in TLR9^−/−^ mice, LSK^+^ (Lin^−^Sca1^+^Kit^+^) cells were purified from WT and TLR9^−/−^ bone marrow by flow cytometry and cultured in vitro. Cocktails of cytokines specific for lymphoid or myeloid cell differentiation were added to the cultures, and the differentiation of B cells (B220^+^) and myeloid cells (CD11b^+^) was assessed by flow cytometry. We found that TLR9^−/−^ LSK^+^ cells produced more CD11b^+^ myeloid cells but fewer B220^+^ lymphoid cells than WT cells during in vitro differentiation (Fig. 6b). To further verify these findings in vivo, a single dose of 5-FU was injected into TLR9^−/−^ and WT mice, and bone marrow cells were analyzed by flow cytometry 12 days after treatment. Consistent with earlier results, TLR9^−/−^ mice showed fewer B220^+^ cells but a significantly increase in total CD11b^+^ myeloid cells and monocyte/macrophages (CD11b^+^CD115^+^ and CD11b^+^Ly6c^hi^ cells) than the WT mice after challenge with 5-FU (Fig. 6c).

Since we showed that the transplantation of TLR9^−/−^ bone marrow cells alone induced significant bone loss and a low level of systemic inflammation in recipient mice (Fig. 2j–p), we next examined the phenotypes of immune cells in chimeric mice transplanted with TLR9^−/−^ or WT bone marrow cells (KO^chim^ versus WT^chim^ mice) to investigate whether myeloid-biased hematopoiesis plays a role in bone loss in TLR9^−/−^ mice. There were no differences in the frequencies of splenic T cell populations between KO^chim^ and WT^chim^ mice (Fig. S3l). KO^chim^ mice showed fewer CD4^+^ T cells in their bone marrow, and no significant difference in the proportion of splenic or bone marrow CD8^+^ T cells was found between the two groups (Fig. S3l, m). However, the counts of bone marrow myeloid progenitor cells and OCPs were significantly increased in KO^chim^ mice (Fig. 6d). KO^chim^ mice also presented more myeloid cells but fewer B cells in their spleen and bone marrow (Figs. 6d, e and S3l, m). These findings indicate that the increased myelopoiesis observed in the bone marrow of TLR9^−/−^ mice was transferable and was the major cause of the lower bone mass and low-level inflammation status in the receiver mice, further suggesting that myeloid-biased hematopoiesis plays an important role in osteoclastic bone loss in TLR9^−/−^ mice.

Myeloid-biased hematopoiesis is a signature of chronic inflammation and is believed to play a critical role in the pathogenesis of many diseases.^48,49,58^ To investigate whether the expansion of myeloid cells in TLR9^−/−^ mice was related to inflammation, we also examined the changes in myeloid populations in WT and TLR9^−/−^ mice after anti-TNF*α* therapy. We found that the frequencies of CD11b^+^ myeloid cells and Ly6c^hi^ OCPs in the bone marrow of TLR9^−/−^ mice were restored to a similar level to that in WT mice after treatment with an anti-TNF*α* antibody (Fig. S4c). No significant change was found in WT mice after anti-TNF*α* therapy. This result indicates an important role of inflammation in promoting myelopoiesis in TLR9^−/−^ mice. In addition, since we have shown that the altered gut microbiota is the major source of inflammation in TLR9^−/−^ mice and that the elimination of the microbiota significantly lowered the inflammation level, the changes in myeloid cells were also investigated in GF mice. We found that similar numbers of bone marrow CD11b^+^ cells, OCPs (B220^-^CD11b^-^Ly6c^hi^) and splenic macrophages (CD11b^+^F4/80^+^) between GF TLR9^−/−^ and WT mice (Figs. 6f and 4h). Furthermore, after cohousing for 9 weeks, the proportions of myeloid cells and progenitors, including bone marrow CD11b^+^ cells, OCPs, CMPs, GMPs and spleen macrophages, also became similar between cohoused TLR9^−/−^ and WT mice (Fig. 6g and S5n). Similar to other reports,^59^ these results suggest that the gut microbiota affects the balance of hematopoiesis in TLR9^−/−^ mice and that this effect of the gut microbiota on hematopoiesis is likely mediated by the modulation of inflammation in TLR9^−/−^ mice. Taken together, our findings further indicate that inflammation-induced myeloid skewing plays an important role in osteoclastic bone loss in TLR9^−/−^ mice.

### scRNA-seq reveals upregulated Fos and Spi1 expression in TLR9^−/−^ monocyte and osteoclast progenitors

Osteoclasts are derived from monocyte/macrophage progenitors. To explore the molecular mechanism of increased osteoclast differentiation in TLR9^−/−^ mice, the scRNA-seq data of monocytes and macrophages (Clusters 5, 11, 15, 21, 27) were combined for analysis. We found that among the total monocytes/macrophages, the overall expression of Fos and Spi1 (also known as PU.1) was significantly upregulated and the number of RANK^+^ cells was increased in the TLR9^−/−^ group (Fig. S9c). The increase in RANK^+^ cells indicated the existence of more OCPs in TLR9^−/−^ monocytes, which was also shown by flow cytometry analysis (Fig. 6a). In line with other reports,^50,60^ monocyte-specific regulons, including Klf4, Fos, Cebpb and Spi1, were identified by SCENIC (Fig. S9a). The activities of Cebpb, Fos and Spi1, which are important regulators of monocyte activation and differentiation,^60^ were significantly upregulated in TLR9^−/−^ monocytes/macrophages (Fig. S9b). Furthermore, the activity of Cebpd, an important indicator of inflammation, was dramatically increased in TLR9^−/−^ monocytes/macrophages (Fig. S9b), suggesting a possible role of inflammation in TLR9^−/−^ monocyte differentiation and function. Interestingly, SCENIC analysis also revealed that Fos, Cebpd and Spi1 were each other’s transcriptional targets (Fig. S9d). The increased transcriptional network among Fos, Cebpd and Spi1 may play important roles in promoting the differentiation and function of TLR9^−/−^ monocytes by targeting monocyte-related genes (Fig. S9d).

**Fig. 7:**
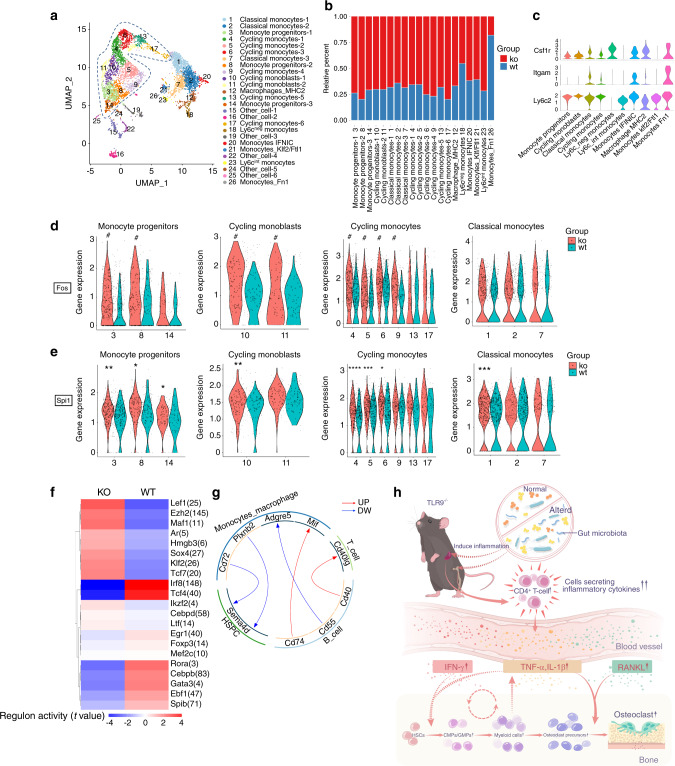
scRNA-seq mapping of bone marrow monocyte/macrophages and T cells. **a**–**e** Mapping of monocyte/macrophages. **a** The reclustered monocyte/macrophages (pool of cluster 5, 11, 15, 21, 27 in Fig. 5a). The immature monocytes are circled with a blue dotted line. **b** Relative percentages of KO and WT cells in each subcluster. *X* axis represents cluster number and cell type. **c** Expression of OCP markers in different cell types. Clusters of the same cell types in **a** were combined for analysis. **d**, **e** Differences of Fos (**d**) and Spi1 (**e**) expression between KO and WT cells in the indicated subclusters. *X* axis represents subcluster numbers. In **d**, the pound sign means significant difference in average expression of Fos with *P* < 0.05 and fold change > 1.5. **f**, **g**. Analysis of bone marrow T cells. **f** SCENIC analysis reveals differences of the regulon activities between TLR9^−/−^ (KO) and wildtype (WT) cells in total T cells. **g** Ligand-receptor predictions among indicated cell types. Red and blue arrows show the upregulated and downregulated ligand-receptor pairs in KO cells, respectively. **h** Proposed model for the mechanism of osteoclastic bone loss in TLR9^−/−^ mice. The TLR9^−/−^ mice exhibit a low-grade systemic inflammation derived from the altered gut microbiota. The systemic inflammation features expansion of CD4^+^ T cells and increased inflammatory cytokines. These cytokines not only stimulate OCPs to differentiate into osteoclasts, but also increase the pool of OCPs by promoting the myeloid-biased hematopoiesis. The dual effect of the inflammatory cytokines significantly promote osteoclastogenesis and bone loss in TLR9^−/−^ mice. Furthermore, increased myeloid cells may also contribute to the maintenance of the chronicity of inflammation, resulting in a feed-forward loop. In **d** and **e**, the asterisks mean significant difference in average gene expression. Wilcoxon test was used to determine the significance in **d** and **e**. *P *<* 0.05, **P < 0.01, ***P < 0.001 and ****P < 0.000 1

Next, we divided all monocytes/macrophages into 26 subclusters, as shown by UMAP embeddings (Fig. 7a). Based on specific gene expression (Fig. S9h), there were 3 clusters of monocyte progenitors (Mpo, Ms4a3, Ifitm1 and Elane), 2 clusters of cycling monoblasts (moderate expression of Mpo, Ms4a3 and Lyz2; enriched for cell cycle genes), 3 clusters of classical monocytes (Lyz2, Lgals3, and Ccr2), 6 clusters of cycling monocytes (enriched for cell cycle genes) and 1 cluster of macrophages (H2-Aa and C1qb). Other small populations of monocytes included Ly6c-intermediate monocytes (moderate expression of Ly6c2), Ly6c-negative monocytes (enriched for Nr4a1 with no expression of Ly6c2), monocytes-IFNIC (IFN-inducible monocytes, enriched for Ifit3 and Irf7), monocytes enriched for Klf2 and Ftl1 (Monocytes_Klf2/Ftl1) and monocytes enriched for Fn1 (Monocytes_Fn1). There were 6 clusters of nonmonocyte cells (Other_cell) that were excluded from further investigations. There were more TLR9^−/−^ cells than WT cells in all subclusters except for Clusters 18 and 26 (Fig. 7b).

Among the subclusters of monocytes/macrophages, we found that cell types including monocyte progenitors (Clusters 3, 8 and 14), cycling monoblasts (Clusters 10 and 11) and cycling monocytes (Clusters 4, 5, 6, 9, 13 and 17), exhibited phenotypes similar to those of OCPs (CD11b^lo/-^Ly6c^hi^CD115^+^)^61^ (Fig. 7c). These cell types mostly represented immature monocytes, as shown by UMAP (circled clusters in Fig. 7a). Interestingly, within these cell types except for Clusters 13, 14 and 17, the expression of Fos was significantly higher in the TLR9^−/−^ group (Fig. 7d). The difference in Fos expression in classical monocytes (Clusters 1, 2 and 7) was not as significant as that in immature monocytes (Fig. 7d). This result is consistent with the identification of higher Fos protein level in TLR9^−/−^ OCPs (Fig. 1i). In addition, higher Spi1 expression was observed in the TLR9^−/−^ monocyte progenitors (Fig. 7e). Taken together, the results indicated that TLR9^−/−^ monocytes showed increased expression and transcriptional activity of Fos and Spi1. Due to the crucial role of Spi1 and Fos in osteoclast commitment, the specific enrichment of Spi1 and Fos in TLR9^−/−^ immature monocytes may constitute a molecular basis for increased osteoclast differentiation.

To further expand our study of TLR9^−/−^ monocytes, Kyoto Encyclopedia of Genes and Genomes (KEGG) enrichment analysis was performed. The results showed that in classical and cycling monocytes, the genes that were upregulated in TLR9^−/−^ cells were mainly involved in lysosome and antigen presentation (Fig. S9e, f). Similarly, in monocyte_IFNIC (IFN-inducible monocytes, Cluster 20), the upregulated genes identified in TLR9^−/−^ cells were involved in infectious diseases, lysosomes, antigen presentation and osteoclast differentiation (Fig. S9g). These results further suggest that TLR9^−/−^ monocytes may be activated by inflammatory stimuli and show increased immune activities for host defense.

### scRNA-seq reveals the activation of T cells in the bone marrow of TLR9^−/−^ mice

Above, we described a trend toward an increase in T cells in the bone marrow of TLR9^−/−^ mice (Fig. 1o and S2d). To probe the molecular characteristics underlying this observation, we assessed the gene expression pattern of bone marrow T cells by scRNA-seq. SCENIC analysis based on total T cells (Cluster 14 in Fig. 5a) showed that TLR9^−/−^ T cells presented increased activity of Tcf7 and Lef1, which are important T cell development factors and are crucial for the differentiation and persistence of memory T cells.^62^ The activity of Foxp3, a signature TF of T regulatory cells (Tregs), was downregulated in TLR9^−/−^ T cells (Fig. 7f). The analysis of cell interactions showed an increase in T-B cell interaction mediated by CD40-CD40L in the bone marrow of TLR9^−/−^ mice (Fig. 7g). The CD40-CD40L interaction stimulates the B cell production of RANKL.^63^ Thus, increased T-B cell interactions mediated by CD40-CD40L ligation may contribute to the increased production of RANKL by TLR9^−/−^ bone marrow B cells.

Then, we further subdivided T cells into 11 distinct clusters, representing 2 clusters of NK T cells (Nkg7 and Klrb1c), CD4^+^ central memory (TCM) T cells (CD4, Ccr7, and Lef1), CD4^+^ effector memory (TEM) T cells and Tregs (S100a4, Tnfrsf4, Icos and Foxp3), CD8^+^ TCM T cells (CD8a, Ccr7 and Lef1), 2 clusters of CD8^+^ TEM T cells (CD8a, Ccl5) and other small populations of T cells, including *γδ* T cells (Id3 and Trgv2) and T cells enriched for ICOS (Fig. S10a, b). There were more TLR9^−/−^ (KO) T cells in all subclusters (Fig. S10c). Gene ontology (GO) enrichment analysis indicated that in CD4 TCM cells (Cluster 3), CD8 TEM (Cluster 5) cells and *γδ* T cells (Cluster 11), KO-upregulated genes were preferentially linked to the positive regulation of the immune response and activation of T cells (Fig. S10d, f). In the CD4 TEM and Treg cluster, KO-upregulated genes were dominantly associated with the IL4 response (Fig. S10f). Notably, we found that in the cluster of CD4 TCM cells (Cluster 3), TLR9^−/−^ cells showed significantly higher expression of Ccr7 (Fig. S10e), an important T cell homing factor.^64^ Since bone marrow T cells mainly consist of migratory memory T cells,^65^ it is possible that the upregulation of Ccr7 promotes the migration of TLR9^−/−^ CD4^+^ T cells to bone marrow, contributing to the increased CD4^+^ T cell number in the bone marrow of TLR9^−/−^ mice. Taken together, these results further support the notion of the expansion and activation of bone marrow T cells in TLR9^−/−^ mice, which may contribute to the inflammatory microenvironment in the bone marrow.

## Discussion

TLRs are recognized as important links between bone and the immune system since the activation of TLRs in innate immune cells induces the release of proinflammatory cytokines that promote osteoclast differentiation.^66^ Although numerous studies have focused on the effects of TLR activation on osteoclasts and bone metabolism, whether TLR signaling is required for normal bone remodeling in vivo is controversial and is not completely understood. Sato et al.^67^ reported that the deletion of Myd88, a critical molecule in TLR signaling, resulted in the suppression of osteoclast differentiation and lower bone mass in mice due to a low bone turnover status. It was recently reported that TLR5 deficiency in mice impairs bone strength but fails to produce changes in trabecular bone mass.^68^ Thus, the role of TLR9 in normal bone remodeling in vivo remains unclear.

In the current study, we showed for the first time that TLR9 deficiency leads to a loss of trabecular bone due to increased osteoclastogenesis. Mechanistically, we demonstrated that chronic systemic inflammation driven by the altered gut microbiota of TLR9^−/−^ mice is a major cause of osteoclastic bone loss in TLR9^−/−^ mice (Fig. 7h). Chronic inflammation promotes osteoclastogenesis via two mechanisms: the production of osteoclastogenic inflammatory cytokines that stimulate OCPs to differentiate into osteoclasts and increases in the levels of OCPs arising from the promotion of myelopoiesis in the bone marrow. These two mechanisms act in concert to increase osteoclastogenesis under chronic inflammatory conditions. Intriguingly, in addition to its role in osteoclastogenesis, myeloid-biased hematopoiesis in TLR9^−/−^ mice also contributes to the maintenance and chronicity of inflammation and, thus, a feed-forward loop between inflammation and myelopoiesis (Fig. 7h).

Similar to postmenopausal bone loss, which is now regarded as a low-grade inflammatory condition characterized by the production of TNF*α* by activated T cells,^10^ inflammation in TLR9^−/−^ mice involves the activation and expansion of splenic and bone marrow T cells, resulting in the upregulation of osteoclastogenic cytokines, including TNF*α*, IL1*β*, IFN*γ* and RANKL, and contributing to bone loss. TNF*α* is a powerful stimulator of osteoclastogenesis. The combination of TNF*α* and IL6 induces the formation of OCLs independent of RANKL.^69^ In addition, IL1*β* upregulates the production of RANKL and acts synergistically with TNF*α* in stimulating osteoclastogenesis.^70^ IFN*γ* further activates T cells and induces T cell-derived RANKL and TNF*α* but suppresses OPG production in B cells.^63^ Although TNF*α* may be the key mediator, all of the increased inflammatory cytokines are likely to act synergistically in promoting bone loss in TLR9^−/−^ mice.

Increasing evidence has indicated that TLR9 suppresses inflammation in some disease models, although this remains controversial. Our work supports these findings by showing systemic chronic inflammation in TLR9^−/−^ mice with a standard C57BL6/J background. Our results may reveal the basis of the exacerbated inflammation observed in disease-prone TLR9^−/−^ models in previous studies.^23–28^ Similar to our findings, Tilstra et al.^71^ observed that the specific deletion of TLR9 in B cells resulted in a greater number and increased activation of CD4^+^ T cells, along with fewer CD19^+^ B cells in the spleen of MRL/lpr mice. The authors also concluded that B cell-intrinsic TLR9 is critical to restrain inflammation in their mouse model. Although the causal role of TLR9^−/−^ B cells in the development of inflammation needs to be further studied, our work highlights the importance of TLR9 in restraining inflammation and preventing bone loss under normal conditions.

A major finding of our current study is that chronic inflammation may also promote osteoclastogenesis and bone loss via myeloid-biased hematopoiesis. Increased myelopoiesis is a hallmark of many chronic inflammatory disorders. Although the role of myelopoiesis during inflammation has been appreciated in studies of acute and chronic inflammatory conditions, including myocardial infarction, atherosclerosis, aging and cancer,^48,49^ the contribution of inflammation-associated myelopoiesis to osteoclastogenesis and bone remodeling remains to be elucidated. HSPCs in the bone marrow sense inflammatory stimuli and adapt through increased proliferation and skewing toward the myeloid lineage. Due to the myeloid origin of osteoclasts, myeloid skewing may also provide a larger OCP pool. Indeed, in the context of chronic inflammation, we observed myeloid-biased hematopoiesis with an increase in OCPs in the bone marrow of TLR9^−/−^ mice and chimeric mice that received TLR9^−/−^ bone marrow cells. This result also reveals the underlying reason for the greater osteogenic potential of TLR9^−/−^ BMNCs during in vitro osteoclastogenesis.

Since inflammatory factors, including TNF*α*, IL1*β*, IL6 and IFN*γ*, work together and play a central role in the adaptation of HSPCs to inflammation,^49,72^ it is likely that chronic exposure to systemically elevated inflammatory cytokine levels is the major cause of increased myelopoiesis in TLR9^−/−^ mice. The decrease in myeloid cells and OCPs observed in TLR9^−/−^ mice after anti-TNF*α* therapy indicates the important role of inflammation in myeloid-biased hematopoiesis (Fig. S4c). The increased transcriptional activity of Cebpd also suggests the occurrence of priming by inflammatory signals in TLR9^−/−^ HSPCs, since Cebpd is recognized as an inflammation indicator and is mainly activated by inflammatory stimuli such as TNF*α*, IL6, IFN*γ*, LPS and IL1*β*.^73^ Additionally, our experiments involving GF and cohoused animal models showed that the differences in the myeloid cells and progenitors between TLR9^−/−^ and WT mice were abolished following the normalization of the inflammatory cytokine levels. These results further suggest the role of inflammation in priming HSPCs in the direction of myelopoiesis in TLR9^−/−^ mice.

It has been proposed that as a result of the adaptation of HSPCs to inflammation, increased myeloid cell numbers further enhance inflammation, thus creating a feed-forward loop that sustains chronic inflammatory conditions.^48^ Our results from the bone marrow chimera (BMC) model support this notion. Since the biased hematopoiesis induced by inflammatory signals is transplantable without further exogenous stimulation in recipients,^74,75^ the BMC is a good model for studying the biology of TLR9^−/−^ hematopoietic cells and their roles in inflammation and bone metabolism. The transfer of myeloid-biased TLR9^−/−^ bone marrow cells not only led to increased myelopoiesis and bone loss but also recapitulated the phenotypes of an enlarged spleen and elevated inflammatory cytokine levels in recipient mice (Fig. 2j–p). These findings suggest the possible mechanism of a myelopoiesis-inflammation feed-forward loop in TLR9^−/−^ mice (Fig. 7h), thus revealing a novel role of myeloid skewing in promoting bone resorption during chronic inflammation.

Recently, scRNA-seq has come to be considered a novel and powerful tool for defining myelopoiesis in the context of diseases in mouse models.^49^ In our study, we used this technique to investigate the molecular traits of the cells involved in this phenomenon at the cellular level. In acute or chronic inflammation, HSCs can bypass steps of the traditional hematopoietic hierarchy via direct differentiation into myeloid progenitor cells.^76^ Taking advantage of scRNA-seq in the analysis of the cell developmental trajectory, we observed faster lineage commitment and more rapid monocyte differentiation during the development of TLR9^−/−^ bone marrow cells. Additionally, evidence shows that HSPCs acquire transcriptional memory upon exposure to inflammatory stimuli.^77^ By scRNA-seq, we also found that TLR9^−/−^ HSPCs were skewed toward the myeloid lineage because of the upregulation of myeloid signature genes, the activation of myeloid-related TFs and the downregulation of lymphoid development genes. Specifically, Fos mRNA was consistently upregulated in TLR9^−/−^ HSPCs and subclusters of these cells (HSC, CMP, GMP and MDP) (Figs. 5j–l and S8g). The upregulation of Fos favors myeloid and monocyte differentiation^52,78^ and may constitute a molecular basis of the myeloid-biased hematopoiesis observed in TLR9^−/−^ bone marrow cells. In addition, the downregulation of critical lymphoid development genes, including Sox4,^54^ Satb1^79^ and Pax5, in TLR9^−/−^ HSPCs may explain the decrease in B cells in the bone marrow of TLR9^−/−^ mice.

By using the scRNA-seq technique, we found that Spi1 and Fos, which are critical TFs mediating the differentiation of monocytes and osteoclasts, were specifically upregulated in TLR9^−/−^ monocyte progenitors. SCENIC analysis also identified increased activities of Fos and Spi1 in TLR9^−/−^ bone marrow monocytes. These findings may indicate the molecular basis of the inflammation-mediated osteoclastogenesis observed in our model. Fos and Spi1 are both downstream targets of inflammatory signals. Increased activity of Cebpd and the transcriptional network among Cebpd, Fos and Spi1 suggest the activation of inflammatory signaling in TLR9^−/−^ bone marrow monocytes. TNF*α* is a potent inducer of Spi1 in myeloid progenitors and HSCs.^80^ It has been reported that Spi1 autoregulates its own promoter and inflammatory signals to activate the Spi1 protein, which also induces the upregulation of Spi1 expression in monocytes.^81^ In the early stage of osteoclast commitment, Spi1 directs the expression of the CSF1 receptor,^82^ which is upregulated in TLR9^−/−^ osteoclast precursors as well (Fig. 1i). A previous in vitro study^34^ showed that the TLR9 ligand inhibits osteoclastogenesis by promoting the degradation of Fos. However, the increased expression of Fos may be a result of inflammation since exogenous signals such as LPS and TNF*α* induce the expression of Fos in monocytes/macrophages.^83^ Fos is a critical factor in osteoclast differentiation. A lack of Fos abolishes osteoclastogenesis and induces osteopetrosis in mice,^84^ while the overexpression of Fos reverses the OPG-mediated suppression of osteoclastogenesis.^85^ In summary, prolonged exposure to inflammatory signals in TLR9^−/−^ mice may sustain higher expression and transcriptional activity of Fos and Spi1 in progenitors during myelopoiesis and monocyte differentiation. Increased expression and activation of Fos and Spi1 in turn contribute to the increased osteoclast commitment observed in TLR9^−/−^ mice.

Alterations in the composition of the gut microbiome have been associated with a number of chronic conditions, including IBD, metabolic disorders, cancer and skeletal diseases.^86^ The immune system shapes the composition of the gut microbiota, and there are reciprocal interactions between gut commensal species and the immune system. It has been reported that the genetic ablation of innate immune molecules such as TLR5, Myd88 and Nlrp6 results in the alteration of the gut microbiota, which can either induce or protect against diseases through the modulation of the immune system and inflammation.^41,42,87^ However, the role of the gut microbiota in TLR9-associated diseases has not been extensively studied. In the current work, we showed that TLR9 deficiency induces specific changes in the gut microbiota, especially increases in certain gram-negative bacteria, and that the altered microbiota plays an important role in mediating systemic chronic inflammation and the resulting bone loss in TLR9^−/−^ mice. (Fig. 7h). Inflammation is a critical link between the gut microbiota and bone.^7,10,11^ In GF mice, sex steroid deficiency fails to increase osteoclastogenic cytokine production and causes trabecular bone loss.^10^ We identified a similar pathway in our model by showing that microbiota depletion or cohousing reduced inflammation levels, decreased bone resorption and restored bone mass in TLR9^−/−^ mice. Furthermore, it has been suggested that the microbiota regulates the HSPC pool and the differentiation of these cells toward myeloid lineages through the upregulation of inflammatory cytokine levels.^59^ As we also found that inflammation contributes to the myeloid-biased hematopoiesis and expansion of OCPs, the decreased inflammatory burden may explain why the frequencies of myeloid cells and OCPs in GF and cohoused TLR9^−/−^ mice were restored to the same levels as in their WT counterparts.

It has been suggested that microbial dysbiosis promotes intestinal and systemic inflammation.^88,89^ In addition to the increase in inflammatory cytokines in the circulation, we identified subclinical chronic inflammation in the gut environment of TLR9^−/−^ mice, which was presumably caused by the alteration of the gut microbiota. Increased gut inflammation may induce bone loss due to chronic systemic inflammation.^90^ Patients with Crohn’s disease (CD) exhibit increased levels of proinflammatory cytokines, including IL-6, TNF*α*, IL-1 and RANKL, and benefit from anti-TNF*α* therapy targeting gut inflammation and bone density.^6^ These observations are in line with our data and further suggest that the gut may be the origin of the systemic chronic inflammation inducing bone loss in TLR9^−/−^ mice.

In conclusion, our work provides novel insights into a fundamental regulatory role of TLR9 in maintaining the homeostasis of inflammation, hematopoiesis and bone metabolism under physiological conditions. Since the alteration of the gut microbiota was identified as an important cause of inflammation and bone loss in TLR9^−/−^ mice (Fig. 7h), the current study also implies that efforts to manipulate gut commensal species may be explored as an alternative way to prevent inflammatory bone loss in the future.

## Materials and methods

### Animal experiments

#### Mice

TLR9^−/−^ C57BL6/J and WT C57BL6/J mice were kindly provided by Prof. Li Wen (Yale University School of Medicine, New Haven, CT) and were described in previous reports.^36,37^ Mouse tail DNA extraction and PCR genotyping were performed using the REDExtract-N-AMP tissue PCR kit (Sigma–Aldrich) following the manufacturer’s recommended protocol. The primers used were as follows: forward, 5′-CCTGAAGTCTGTACCCCGTT-3′; reverse, 5′-TCTGGGCTCAATGGTCATGT-3′. An amplicon of 317 bp was generated from the WT allele, and a 516 bp product was amplified from the disrupted allele. TLR9^−/−^ and WT mice were bred in separate cages and fed normal chow. The Rag1 knockout mice (Rag1^−/−^) used for the adoptive transfer experiment were obtained from the Model Animal Research Center of Nanjing University. All procedures were carried out in accordance with approved IACUC animal protocols at our institute (Shanghai Jiaotong University-Affiliated No. 6 People’s Hospital).

#### Preparation of mouse serum bone marrow supernatants

For serum collection, blood samples were drawn from the ophthalmic vein of mice. The samples were then centrifuged at the maximum speed on a table-top centrifuge, and supernatants were collected as serum samples. For bone marrow supernatants, both ends of the mouse femurs were removed, and the diaphyseal bone was placed upright in an Eppendorf tube. After centrifugation at 850 × *g* for 30 s, the cell pellet and its supernatant was resuspended with 80 μL PBS and centrifuged again at 2 300 × *g* for 1 min. Then, the supernatants were collected and transferred to a new tube before storage at −80 °C

#### Generation of GF mice and cohousing

GF TLR9^−/−^ and WT mice were rederived via hysterectomy and fostered by an axenic mother. The mice were then maintained in gnotobiotic facilities for 8 weeks before being sacrificed for further analysis. For cohousing, the same number of newly weaned (3 weeks old) sex-matched TLR9^−/−^ and WT mice were placed in a new cage and fed for an additional 9 weeks before being sacrificed for further analysis.

#### Adoptive transfer of splenocytes to Rag1^−/−^ mice

Six-week-old male Rag1^−/−^ mice (C57BL6/J background) were randomly distributed into two groups to be used as recipients of TLR9^−/−^ or WT splenocytes (KO_Rag1^−/−^ and WT_ Rag1^−/−^ group). A total of 10^7^ splenocytes that had been freshly harvested from male TLR9^−/−^ or WT mice were injected into Rag^−/−^ mice intravenously (ophthalmic vein) in a final volume of 200 μL. After 6 weeks, the mice were sacrificed for further analysis.

#### 5-Fluorouracil (5-Fu) treatment

5-Fu (Sigma–Aldrich) was prepared in normal saline at a concentration of 10 mg·mL^−1^. Then, a single dose of 5-Fu (1.5 mg per 10 g body weight) was injected into 8-week-old male TLR9^−/−^ and WT mice intraperitoneally (i.p.)), and the mice were sacrificed 12 days after treatment.

#### Generation of bone marrow chimeras

Six-week-old male C57BL6/J mice were exposed to a lethal dose of X-ray irradiation (10 Gy, rs2000 irradiator, Rad Source Technologies) before bone marrow transplantation. Within 6 h after irradiation, 10^7^ donor bone marrow cells (from male TLR9^−/−^ or WT mice) were injected intravenously (ophthalmic vein) into each irradiated mouse in a final volume of 200 μL. Recipient mice were sacrificed for further analysis 6 weeks after bone marrow transplantation.

For the genotyping of bone marrow chimera mice, a separate experiment was performed at the same time by transferring bone marrow cells from WT male donors to lethally irradiated female WT mice. Three weeks after cell transfer, bone marrow cells from the female recipient mice were collected, and total DNA was extracted using a DNeasy Blood & Tissue Kit (Qiagen). PCR was performed using the Ex-Taq enzyme (Takara) following the kit instructions. The following primers were used: forward: 5′-GATGATTTGAGTGGAAATGTGAGGTA-3′, and reverse: 5′-CTTATGTTTATAGGCATGCACCATGTA-3′. Initial denaturation was performed at 98 °C for 1 min. Amplification was performed for 35 cycles, in which denaturation, annealing and extension were conducted at 98 °C (15 s), 55 °C (30 s) and 72 °C (60 s), respectively. Final elongation was performed at 72 °C for 10 min. The PCR products were subjected to electrophoresis together with a 100 bp DNA ladder and visualized with ethidium bromide staining under UV illumination. Amplification from the Y chromosome yielded a 280 bp product, while amplification from the X chromosome yielded two bands with sizes of approximately 480 bp and 660 bp.^91^

#### Anti-TNFα therapy in vivo

For the delivery of TNF*α* neutralizing antibodies (MP6-XT3, 1 mg·mL^−1^, Invitrogen) or the IgG1 kappa isotype control (eBRG1, 1 mg·mL^−1^, eBioscience), a total of 200 μL of the undiluted TNFα antibody or IgG was loaded into each Alzet minipump. The pumps were implanted subcutaneously into 6-week-old male TLR9^−/−^ or WT mice, and the treatment continued for a total of 6 weeks.

#### Bone density measurements

Femurs and the L3 lumbar vertebrae were stripped of soft tissue, stored in 75% EtOH at 4 °C, and scanned using a μCT instrument (SkyScan 1176). The scanning voxel size was 9 × 9 × 9 μm^3^. The X-ray tube potential was 50 kV, with a current of 450 µA. The integration time was 520 ms. The rotation step was 0.4° for 180° scanning. The image analysis was performed using CTAn micro-CT software version 1.13 (Bruker). We applied a threshold value of 75 (grayscale index) for all femur and lumbar trabecular bone analyses. A threshold value of 110 (grayscale index) was used for all cortical bone analyses. Volumetric regions for trabecular analyses, selected within the endosteal borders of the distal femoral metaphysis to include the secondary spongiosa located 1 mm from the growth plate and extending 1.8 mm (200 sections) proximally, were scanned at a 9 μm resolution. For vertebrae, the entire trabecular region excluding the primary spongiosa (300 μm below the cranial and above the caudal growth plate) was analyzed. A cortical bone region 600 μm long at the mid-diaphysis of the femur (extending 300 μm proximally and distally from the diaphyseal midpoint between the proximal and distal growth plates) was quantified. No cortical analyses were performed on vertebrae. The collected data included bone mineral density (BMD), the bone volume/total volume fraction (BV/TV), trabecular number (Tb.N), trabecular thickness (Tb.Th) and trabecular separation (Tb.Sp). Cortical bone indices including cortical thickness (Ct.Th), total cross-sectional area (Tt.Ar) and cortical area (Ct.Ar) were also quantified.

#### Bone histomorphometry analysis

Dissected femurs were fixed in 4% paraformaldehyde for 48 h and decalcified using 10% EDTA (pH = 7.2) at 4 °C for approximately one week. The specimens were then embedded in paraffin and sectioned at a thickness of 4 µm. TRAP staining was performed to analyze osteoclasts. Immunohistochemistry staining using an anti-osteocalcin antibody (Abcam, ab93876) was used for the analysis of osteoblasts. Measurements were performed in an ~2.5 mm^2^ area of distal femoral cancellous bone containing only secondary spongiosa, located 0.5–2.5 mm proximal to the epiphyseal growth cartilage. Calcein labeling was used for dynamic histomorphometry analysis. Mice were injected intraperitoneally with 30 μg·g^−1^ body weight calcein (Sigma-Aldrich) on Day 1 and Day 7 before being sacrificed. Their bones were then fixed, dehydrated, embedded with methylmethacrylate and analyzed in unstained sections. The MAR and bone formation rate were calculated by software by applying the interlabel period. Histomorphometry was performed using Bioquant Osteo software (Bioquant). The accepted nomenclature was used to report the results.^92^

### Cell culture and cell-based experiments

#### In vitro osteoclastogenesis assay

Bone marrow cells were first obtained by flushing the mouse femurs and tibias. The cells were cultured overnight in α-MEM (HyClone) containing 10% FBS, 100 U·mL^−1^ penicillin, 100 μg·mL^−1^ streptomycin (Gibco) and 20 ng·mL^−1^ M-CSF (Peprotech) to remove adherent cells. Nonadherent cells were then collected, layered on Ficoll-Paque (GE Healthcare) and centrifuged. BMNCs at the interface were collected and washed twice with ice-cold PBS. For osteoclast differentiation, BMNCs (2.5 × 10^4^ cells per well for 96-well plates or 1.2 × 10^5^ cells per well for 24-well plates) were cultured in α-MEM containing 10% FBS, 100 U·mL^−1^ penicillin, 100 μg·mL^−1^ streptomycin (Gibco), 100 ng·mL^−1^ M-CSF (Peprotech) and 100 ng·mL^−1^ RANKL (Peprotech) for 5 days before TRAP staining or lysis for RNA extraction. The cells were maintained at 37 °C in a humidified incubator under 5% CO_2_, and the medium was changed every other day.

For treatment with a TLR9 antagonist, 0–10 μmol·L^−1^ CpG-ODN 2088 (Invivogen) was added to the cell culture together with RANKL and CSF1 from the beginning of the assay (Day 0). The cells were cultured for 5 days before TRAP staining.

To quantify osteoclast numbers and area, cells were fixed at the end of the assay (Day 5), and tartrate-resistant acid phosphatase (TRAP) staining was performed using an Acid Phosphatase, Leukocyte (TRAP) Kit (Sigma-Aldrich). Large multinuclear cells (≥3 nuclei) were considered mature OCLs. The analyses were performed in triplicate, and the number of OCLs per well was documented for each biological replicate.

### Harvesting of calvarial osteoblasts and osteogenic differentiation

Newborn TLR9^−/−^ or WT mice (2–3 days after birth) were used to harvest calvaria. After sacrifice, the calvaria were dissected and digested five times in α-MEM containing 0.1% collagenase (Roche) and 0.2% dispase (Roche). Cells collected from the last four digestions were pooled together and cultured in α-MEM containing 10% FBS, 100 U·mL^−1^ penicillin, and 100 μg·mL^−1^ streptomycin. Cells showing confluence of 60%–80% were replated at a density of 1 × 10^5^ per well in 12-well plates, and the culture medium was changed to osteogenic differentiation medium (Cyagen) to induce the differentiation of osteoblasts. After 7 days of differentiation, the cells were either fixed for ALP staining or lysed for RNA extraction or the ALP assay (Jiancheng Bioengineering, Nanjing). For the collection of osteoblast culture supernatants, cells were cultured in regular 10% FBS α-MEM for an additional 2 days before collecting the supernatants. All experiments were performed in duplicate, and the number of biological replicates is indicated by the number of dots in each bar plot.

### HSC differentiation

FACS-sorted LSK^+^ cells (5 000 cells per well in 96-well round-bottomed plates) were cultured in X-VIVO-15 medium (Lonza) containing 3% BSA, 100 U·mL^−1^ penicillin, and 100 μg·mL^−1^ streptomycin. For myeloid differentiation, a cytokine cocktail including 20 ng·mL^−1^ IL-3, 20 ng·mL^−1^ IL-6, 50 ng·mL^−1^ SCF and 30 ng·mL^−1^ M-CSF (Peprotech) was used. The lymphoid differentiation cytokine cocktail contained 100 ng·mL^−1^ IL-7, 50 ng·mL^−1^ Fit3L and 50 ng·mL^−1^ SCF (Peprotech). Half of the cytokine-containing culture medium was refreshed every other day. The cells were cultured for a total of 7 or 12 days for myeloid or lymphoid cell differentiation, respectively.

### Transwell coculture of BMNCs and splenocytes

BMNCs were plated in a 24-well or 96-well dish (lower chamber) at a density of 1 × 10^5^ (24-well) or 1.25 × 10^4^ cells (96-well) per well with α-MEM supplemented with 30 ng·mL^−1^ M-CSF and RANKL. For coculture with total splenocytes, Transwell inserts with a 0.4 μm pore size (Corning) were then inserted into a 24-well dish, and TLR9^−/−^ or WT splenocytes were plated in the upper chamber at a density of 1 × 10^5^ cells per well in RPMI 1640 medium supplemented with 10% FBS, 1 μg·mL^−1^ anti-CD3, 5 μg·mL^−1^ anti-CD28, 3 μg·mL^−1^ anti-IgM and 10 μg·mL^−1^ anti-CD40 (Biolegend). For coculture with splenic T or B cells, the HTS Transwell-96 system with a 0.4 µm pore size (Corning) was used. WT and TLR9^−/−^ T or B cells were magnetically separated using a Mojosort Mouse CD4 T Cell Isolation Kit (Biolegend) or a CD19 B Cell Isolation Kit (Biolegend). The cells were then plated in the upper chamber at a density of 4 × 10^4^ cells per well in 10% FBS-RPMI 1640 medium supplemented with 1 μg·mL^−1^ anti-CD3 and 5 μg·mL^−1^ anti-CD28 (for T cells) or 3 μg·mL^−1^ anti-IgM and 10 μg·mL^−1^ anti-CD40 (for B cells). BMNCs and splenocytes were cocultured for 5 days before fixation for TRAP staining.

#### In vitro culture of T cells

Spleen CD4^+^ T cells were magnetically separated using a Mojosort Mouse CD4 T Cell Isolation Kit (Biolegend) according to the manufacturer’s instructions. T cells were seeded in 96-well round-bottomed plates at a density of 1 × 10^6^ cells per well and cultured in RPMI 1640 medium supplemented with 10% FBS, 1 μg·mL^−1^ anti-CD3 and 3 μg·mL^−1^ anti-CD28 for 48 h. The plates were then centrifuged, and the supernatants were collected.

#### In vitro culture of B cells

Spleen and bone marrow CD19^+^ B cells were magnetically separated using a Mojosort Mouse CD19 B Cell Isolation Kit (Biolegend) according to the manufacturer’s instructions. The cells were seeded in 96-well round-bottomed plates at a density of 1 × 10^6^ cells per well supplemented with 10% FBS, 3 μg·mL^−1^ anti-IgM and 10 μg·mL^−1^ anti-CD40 for 48 h. The plates were then centrifuged, and the supernatants were collected.

#### In vitro culture of bone marrow-derived mesenchymal stem cells (BMSCs)

Bone marrow cells were collected by the flushing femurs and tibias of TLR9^−/−^ or WT mice and were cultured in DMEM (HyClone) containing 10% FBS, 100 U·mL^−1^ penicillin, and 100 μg·mL^−1^ streptomycin. After 48 h, nonadherent cells were removed, and fresh medium was added. The adherent spindle-shaped cells were further cultured for 2 days before collecting the supernatants.

#### Flow cytometry

Splenocytes, bone marrow cells, and cells from MLNs and PPs were obtained via routine procedures. For surface marker staining, isolated cells were blocked with an anti-CD16/32 antibody (Biolegend) for 15 min, followed by staining with fluorescence-conjugated antibodies for 30 min at 4 °C in the dark. Specifically, there was no blocking step when cells were stained with anti-CD16/32-FITC. The antibodies listed as below were purchased from Biolegend (the catalog numbers are provided in parentheses): anti-Ly-6C-Pacific Blue (128013), anti-Ly-6G-Pacific Blue (127611), anti-lineage cocktail-Pacific Blue^™^ (133305), anti-CD4-FITC (100405), anti-CD16/32-FITC (101305), anti-CD4-PE (100407), anti-CD19-PE (115507), anti-CD115-PE (135505), anti-CD127-PE (121111), anti-Ly-6C-PE (128007), anti-CD117-PE (105808), anti-CD4-PE/Cy5 (100409), anti-CD45R-PE/Cy5 (103209), anti-CD19-PerCP/Cyannine5.5 (115533), anti-CD34-PerCP/Cyannine5.5 (128607), anti-CD8-PE/Cy7 (100721), anti-CD115-PE/Cy7 (135523), anti-Ly-6G-PE/Cy7 (127617), anti-CD8-APC (100711), anti-CD135-APC (135309), anti -CD127-APC (135011), anti-CD45R-APC (103212), anti-Ly-6A/E-APC (108111), anti-CD69-APC/Cy7 (104525), anti-CD117-APC/Cy7 (105825), anti-CD4-APC/Cy7 (100413), anti-Ly-6A/E-Alexa Fluor^®^ 700 (108142) and anti-F4/80-Alexa Fluor^®^ 700 (123129).

For intracellular staining, splenocytes and bone marrow cells were stimulated with a Cell Activation Cocktail (with Brefeldin A) (Biolegend) for 4 h at 37 °C in a CO_2_ incubator. Then, the cells were washed, and surface staining was performed as described above. Cells were then fixed and permeabilized using fixation buffer (Biolegend) and intracellular staining perm wash buffer (diluted to 1× with deionized water) (Biolegend). Staining was performed by incubation with antibodies for 30 min at 4 °C in the dark. The antibodies used for intracellular staining were purchased from Biolegend and included anti-IFN𝛄-FITC (505806), anti-TNFα-PE (506306) and anti-CD254-PE (510005).

Measurements were performed on a CytoFlex flow cytometer (Beckman Coulter), and 50 000 events were collected for each sample. Flow cytometry analysis was performed using FlowJo software version 10.4. We first gated singlets on the FSC-A/FSC-H plot. Cell debris and dead cells were then excluded on the FSC-A/SSC-A plot, and the remaining cells gated for analysis were regarded as total splenocytes or bone marrow/MLN/PP cells. The gating strategy for each cell type is shown in Figs. S11–13.

#### Fluorescence-activated cell sorting (FACS)

For the sorting of LSK^+^ cells, bone marrow cells were flushed from mouse tibias and femurs, and BMNCs were enriched by Ficoll-Paque gradient centrifugation. The cells were further enriched using a MojoSort Mouse Hematopoietic Progenitor Cell Isolation Kit (Biolegend) before staining with anti-CD117-PE and anti-Ly-6A/E-APC. Stained cells were sorted on the Moflo XDP (Beckman) platform. CD117^+^Ly-6A^+^ cells were gated as targeted cells in purifying mode. Sorted LSK^+^ cells were washed and maintained in X-VIVO-15 medium containing 3% BSA, 100 U·mL^−1^ penicillin, and 100 μg·mL^−1^ streptomycin supplemented with 50 ng·mL^−1^ SCF overnight before stimulation with cytokine cocktails. The gating strategy for HSPCs is illustrated in Fig. S13.

#### Cell proliferation assay

The experiment was performed using a cell proliferation ELISA BrdU kit (Roche) following the manufacturer’s instructions. Briefly, cells were routinely cultured in flat-bottomed 96-well plates. Before measurements, BrdU was added to the culture medium, and the cells were incubated for another 4 h. The culture medium was then removed, and the cells were sequentially incubated with FixDenat solution, anti-BrdU-POD working solution and substrate solution. When color development was sufficient, the absorbance was measured in a plate reader (BioRad).

#### Real-time PCR

Total RNA was extracted using an RNeasy^®^ Mini Kit (Qiagen) and was reverse transcribed using PrimeScript^™^ RT Master Mix (Takara, RR036A). Real-time PCR was performed using SYBR Premix Ex Taq^™^ II (Takara, RR820L), and samples were run on the ABI HT7900 platform (Applied Biosystems). Amplification was performed for 40 cycles, during which denaturation was performed at 95 °C for 5 s, and annealing and extension were performed at 60 °C for 34 s. A melting curve stage was added to check primer specificity. The relative expression of targeted genes was analyzed using the 2^−∆∆CT^ calculation method. Primers were synthesized by Sangon Biotech company (Shanghai) and are listed in Table S1.

#### Western blot analysis

Cell protein lysates were harvested using regular lysis buffer (Beyotime) supplemented with a Protease Inhibitor Cocktail (Cell Signaling Technology, 1:100). Protein samples were separated by SDS/PAGE and transferred to PVDF membranes (Millipore). The membranes were blocked and then incubated with primary antibodies at 4 °C overnight with gentle agitation. Incubation with HRP-conjugated secondary antibodies was performed at room temperature for 1 h. Protein bands were detected using the Pierce^®^ ECL Western Blotting Substrate (Thermo Fisher Scientific) and visualized with a digital imaging system (BioRad). All primary and secondary antibodies except for anti-RANK (Santa Cruz, catalog no. sc-390655) were obtained from Cell Signaling Technology and included the following(catalog numbers are included in the brackets): anti-GAPDH (5174S), anti-c-Fos (2250S), anti-NFATc1 (8032S), anti-M-CSF receptor (3152S), HRP-linked anti-mouse IgG (7076S) and HRP-linked anti-rabbit IgG (7074P2).

#### Enzyme-linked immunosorbent assay (ELISA)

The concentrations of CTX, P1NP, RANKL, OPG, TNF*α*, IL1*β*, IL17, IL6, IFN*γ*, Lcn2, LPS and IgA were measured using commercial ELISA kits (Jianglai Biological, Shanghai) following the manufacturer’s instructions. Briefly, working standards and diluted samples were prepared and added to each well. The plates were sealed and incubated for 1 h at 37 °C. After washing, 100 μL enzyme-labeled reagents was added, and the plates were incubated for another 1 h at 37 °C. TMB substrates were then added, and the plates were incubated for 15–30 min at 37 °C, followed by the addition of the stop solution. The plates were read at 450 nm within 5 min.

#### Single-cell RNA-seq analysis

BMNCs from 2 WT and 2 TLR9^−/−^ mice were profiled in two independent experiments (each experiment included samples from 1 WT and 1 TLR9^−/−^ mouse). Single-cell RNA-seq of the 5′ end transcriptome was conducted using the 10X Chromium Single Cell Platform (10X Genomics). The generation of gel beads in emulsion (GEMs), barcoding, GEM-RT clean-up, complementary DNA amplification and library construction were all performed following the manufacturer’s protocol. Qubit was used for library quantification before pooling. The final library pool was sequenced on the Illumina NovaSeq 6000 platform using 150-base-pair paired-end reads. Sequencing data were processed using Cell Ranger software (10X Genomics).

#### Preprocessing, clustering and pathway analysis of scRNA-seq data

Raw sequencing data were demultiplexed, aligned, and counted via Cell Ranger pipelines. The Cellranger aggr command was used to combine the sequencing data from multiple libraries with the mapped sequencing depth.

After aggregation of samples from TLR9^−/−^ and WT cells with mapped sequencing depth, the R package Seurat was used for gene and cell filtration, normalization, principal component analysis (PCA), variable gene identification, clustering analysis, and t-distributed stochastic nearest neighbor embedding (tSNE). Analyses were performed with the default parameters. Cells expressing < 200 or >10 000 genes were filtered out to exclude noncell or cell aggregates. Cells with s > 0.10% mitochondrial genes were also filtered out. For UMI normalization, only genes with at least one UMI count detected in at least one cell were retained for analysis. Library size normalization was performed for each cell. The corrected-normalized gene-barcode matrix was used as the input for dimension reduction and clustering analysis.

PCA was performed for dimension reduction. The first 30 principal components were used for clustering analysis. Clusters were visualized with tSNE and UMAP embeddings. The visualization of gene expression was conducted with Seurat functions. Markers for a specific cluster against all remaining cells were identified with the FindAllMarkers function. DEGs (*P* < 0.05 between 2 identities) were identified with the FindMarkers function.

GO and KEGG pathway analyses were performed with the marker genes of each cluster identified by the FindAllMarkers function or enriched genes identified by the FindMarkers function with a fold change>1.5 on the DAVID (database for annotation, visualization and integrated discovery) website and were then plotted with the R package ggplot2.

For the subclustering of the indicated populations, the raw data of the cells were retrieved from the Seurat object containing an aggregated expression matrix for the creation of a new and separate Seurat object. Similar gene filtration, PCA, clustering, tSNE, UMAP embedding and pathway enrichment analyses were then performed.

#### Pseudotime trajectory analysis

Pseudotime trajectory analysis was performed using the R package monocle (version 2.14.0) with the default settings. The DDRTree method was utilized for dimension reduction and cell ordering along the pseudotime trajectory. Branch analysis was performed with branch expression analysis modeling. To present the genes showing significant differences (taken from the first 100 genes showing significantly changes according to the *P* value) at a branch point, 4 gene blocks were chosen according to the distinct patterns of gene expression changes leading to the 2 different cell states.

#### Ligand–receptor cellular communication analysis

To infer potential ligand–receptor interactions between the two cell types, we adopted the method used in CellPhoneDB (https://www.cellphonedb.org). Mouse orthologs of human ligand–receptor pairs compiled by Efremova et al. ^93^ were used for analysis. Enriched receptor–ligand interactions between two cell types were predicted based on the expression of a receptor by one cell type and a ligand by another cell type using our scRNA-seq data.

#### SCENIC analysis

We used SCENIC for gene regulatory network analysis. In brief, we generated coexpression networks of our scRNA-seq data via GRNBoost2. We then utilized the Python SCENIC package to generate cell regulatory networks from our sc-RNA seq data, with the mouse mm10 genome for cis-regulatory analysis. We used two gene-motif rankings: 10 kilobases around the transcription start site (TSS) or 500 base pairs (bp) upstream and 100 bp downstream of the TSS, which were obtained from https://resources.aertslab.org/cistarget/.

#### 16S rRNA seq

Fresh feces were collected from the mouse colon and cecum in sterile tubes. Bacterial genomic DNA was extracted from feces using commercial kits (Magen). The V4 region of the 16S rRNA gene was amplified, and validated libraries were used for sequencing on the Illumina MiSeq platform. Raw reads were filtered, and tags were added to paired-end reads, followed by clustering into operational taxonomic units (OTUs). Then, representative OTU sequences were taxonomically classified and trained using the Greengenes database (v201305). Alpha and beta diversity were estimated using MOTHUR (v1.31.2) and QIIME (v1.8.0) at the OTU level. PCoA was performed by using QIIME (v1.8.0). Bar plots of different classification levels were generated, and significant species were determined by R (v3.4.1) based on the Wilcoxon test. LEfSe cluster analysis and LDA were conducted by LEfSe.

#### Statistical analysis

Comparisons between two groups were performed using two-tailed Student’s *t* tests unless otherwise specified. The Mann–Whitney *t* test (also known as the Wilcoxon rank-sum test) was used for nonparametric analysis. Comparisons between three or more groups were performed using one-way analysis of variance (ANOVA) or two-way ANOVA (for the anti-TNF*α* therapy experiment) with multiple comparison tests (Tukey’s test). Statistical significance was determined using GraphPad Prism Software (v8.2.1), and *P* values < 0.05 were considered statistically significant (*P < 0.05; **P < 0.01; ***P < 0.001; ****P < 0.000 1).

### Resource availability

The scRNA-seq data can be accessed from the GEO database (accession no. GSE166366). The 16S rRNA-seq data are available from the Sequence Read Archive (SRA) under accession numbers PRJNA693037 and PRJNA693105.

## Supplementary information

Supplemental material

## Data Availability

The scRNA-seq data can be accessed from the GEO database (accession no. GSE166366). The 16 S rRNA-seq data are available from the Sequence Read Archive (SRA) under accession numbers PRJNA693037 and PRJNA693105.
