# Correction: Toll-like receptor 9 deficiency induces osteoclastic bone loss via gut microbiota-associated systemic chronic inflammation

**DOI:** 10.1038/s41413-022-00221-0

**Published:** 2022-07-07

**Authors:** Peng Ding, Qiyuan Tan, Zhanying Wei, Qiyu Chen, Chun Wang, Luyue Qi, Li Wen, Changqing Zhang, Chen Yao

**Affiliations:** 1 Department of Orthopedic Surgery, Shanghai Jiaotong University affiliated Sixth People’s Hospital, Shanghai, China; 2 Department of Endocrinology and Metabolism, Shanghai Jiaotong University affiliated Sixth People’s Hospital, Shanghai, China; 3 Department of Osteoporosis and Skeletal Disorders, Shanghai Jiaotong University affiliated Sixth People’s Hospital, Shanghai, China; 4 Department of Endocrinology and Metabolism, Second Affiliated Hospital of Soochow University, Suzhou, China; 5 Section of Endocrinology, Department of Internal Medicine, Yale University School of Medicine, New Haven, USA

**Keywords:** Bone, Pathogenesis

Correction to: *Bone Research* 10.1038/s41413-022-00210-3, published online 27 May 2022

Following publication of the original article [1], the authors regretted to find misinterpretation of data in Fig. 5e, f and Fig. S8b-d. These plots actually showed pseudotime gene expression analysis in the whole bone marrow cells in KO and WT mice, not in the specific clusters as indicated in the original paper. Therefore, Fig. 5e and Fig. 5f showed identical results and so was Fig. S8b–d. To correct this mistake, we deleted the duplicate figures and rearranged the panels in Fig. 5e, f and Fig. S8b–d. The correspondent text (Lines 10-13 of the right column in page 9) and legends (page 10) were also corrected. Since the genes analyzed in these plots were known markers specific for monocyte development, the pseudotime gene analysis among the entire bone marrow cells also strongly supported our conclusion that there were increased myelopoiesis and more rapid monocyte development in the TLR9-/- bone marrow. Although this correction does not affect the results and conclusion of the above paper, all the authors agree to correct this negligence, and feel sorry and sincerely apologize for all the inconvenience it caused.
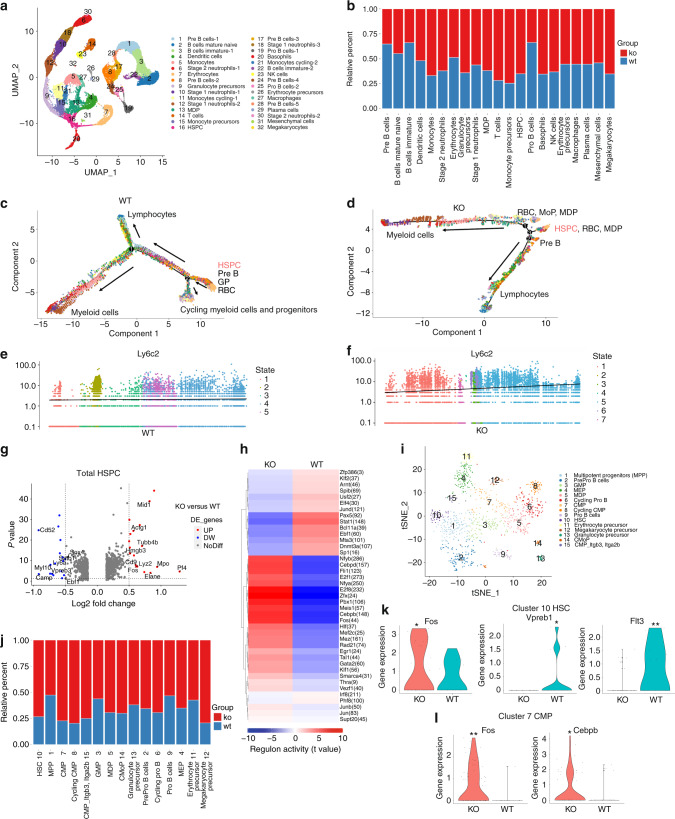


The original article [1] has been corrected.

## Supplementary information

Supplementary Fig. 1
