# Supplementary material for: Toll-like receptor 9 deficiency induces osteoclastic bone loss via gut microbiota-associated systemic chronic inflammation
Source: Bone Res. 2022 May 27;10:42. doi: 10.1038/s41413-022-00210-3 (PMC9142495; doi:10.1038/s41413-022-00210-3)
Supplement: Supplementary file 1 — Supplemental material [file 41413_2022_210_MOESM1_ESM.pdf]

Supplementary Fig. 1

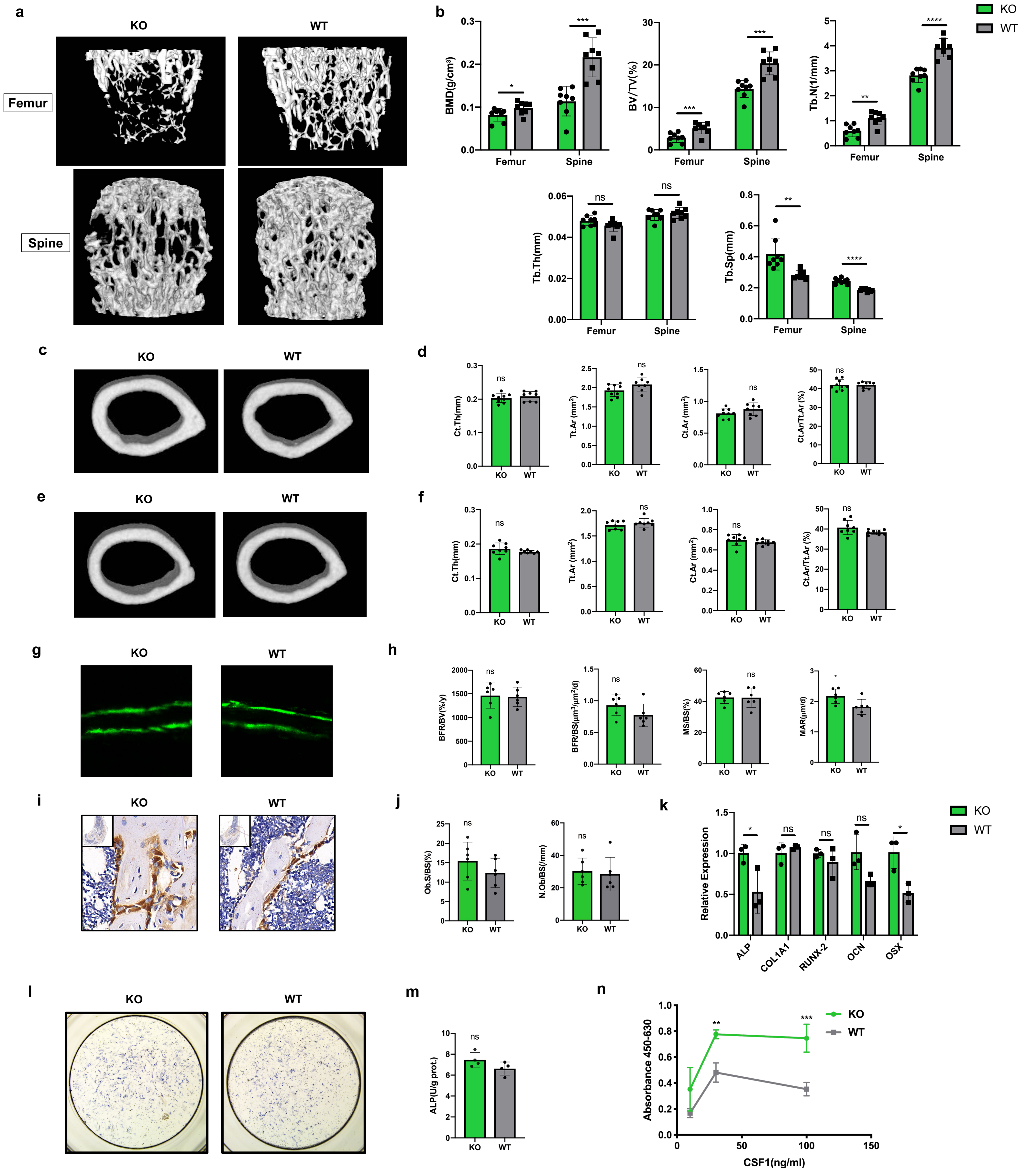

Fig. S1. TLR9$^{-/-}$ mice had low bone mass and increased osteoclastogenesis

a-b Trabecular bone microarchitecture of femurs and vertebrae (3rd lumber spine) of 8-week-old female TLR9$^{-/-}$ (KO) and wildtype (WT) mice. **a** Representative 3-dimensional μCT reconstructions of examined femurs and L3 vertebrae from each group. **b** Quantifications of femur and spine BMD, BV/TV, Tb. N, Tb.Th and Tb.Sp. n=8 per group in all panels. **c-d** Cortical bone of femurs from 8-week-old male KO and WT mice. **c** Representative 3-dimensional μCT reconstructions. **d** Quantifications of Ct.Th, Tt.Ar, Ct.Ar and Ct.Ar/Tt.Ar. n=9 and 8 in KO and WT group, respectively. **e-f** Cortical bone of femurs from 8-week-old female KO and WT mice. **e** Representative 3-dimensional μCT reconstructions. **f** Quantifications of Ct.Th, Tt.Ar, Ct.Ar and Ct.Ar/Tt.Ar. n=8 per group. **g-h** Representative fluorescent images of calcein labeling (**g**) and dynamic indices of bone formation (**h**). n=6 per group in all panels. **i-j** Representative immunohistochemistry images of osteocalcin labeled osteoblasts (**i**) and static indices of bone formation (**j**). n=6 per group in all panels. Eight-week-old male and female mice (sex-matched between KO and WT group) were used in the histomorphometric analysis in **h** and **j**. **k** qPCR analysis of osteoblast-specific genes' expression in cultured KO and WT calvarial osteoblasts. **l-m** Representative ALP staining images (**l**) and quantification of ALP expression using ALP activity assay (**m**) after *in vitro* osteoblast differentiation using calvaria-derived osteoblasts from TLR9$^{-/-}$ and wildtype pups. n=4 per group in **m**. **n** Proliferation of early osteoclast progenitors (OCPs, adherent cells on day 2 of in vitro osteoclastogenesis assay). OCPs harvested from 8-week-old male mice were serum starved for 6 hours before stimulated by serial concentrations of CSF1 in 1% FBS alpha-MEM. Results were determined by BrdU incorporation ELISA assay. n=3 for each concentration. Error bars represent the s.d. *$p < 0.05$, **$p < 0.01$, ***$p<0.001$, ****$p<0.0001$ and ns $p>0.05$; statistical significance was determined using an unpaired two-tailed t-test.

Supplementary Fig. 2

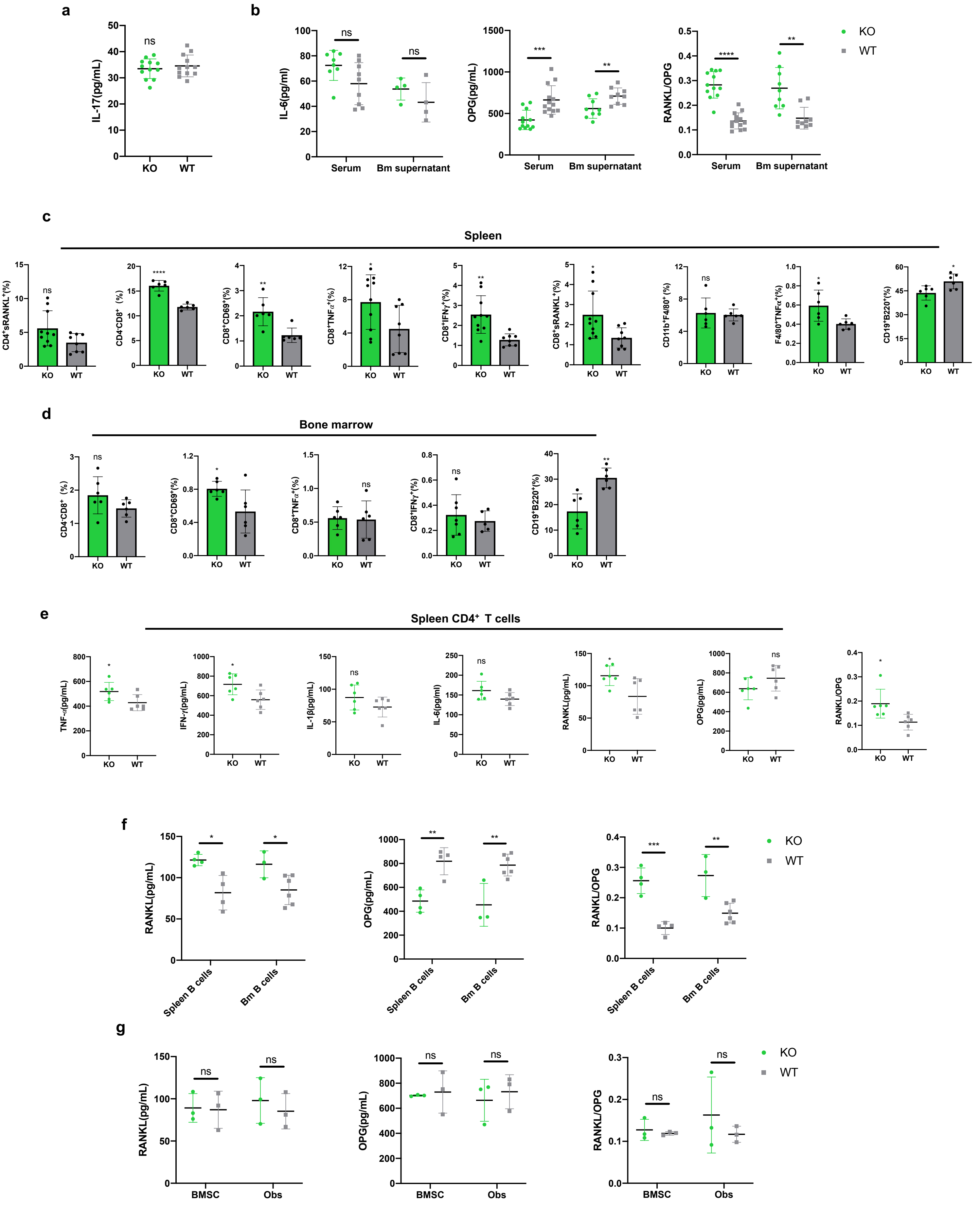

Fig. S2. TLR9$^{-/-}$ mice exhibited chronic systemic inflammation.

a Circulating levels of IL17 in TLR9$^{-/-}$ and wildtype mice. n=12 per group. **b** Serum and bone marrow supernatant concentrations of IL6, OPG and ratio of RANKL/OPG in KO and WT mice. n=4-13 per group. **c-d** Proportions of T, B cell and macrophage populations in the spleen (**c**) and bone marrow (**d**) of KO and WT mice analyzed by flow cytometry. n=6-10 per group. The numbers in represent the frequencies in total splenocytes or bone marrow cells. **e** Inflammatory cytokine levels in culture supernatant of spleen CD4^{+} T cells. n=6 per group in all panels. **f** RANKL, OPG and ratio of RANKL/OPG levels in the culture supernatant of spleen and bone marrow B cells. n=3-6 per group. **g** RANKL, OPG and ratio of RANKL/OPG levels in the culture supernatant of bone marrow mesenchymal stem cells (BMSC) and calvarial osteoblasts (Obs). n=3 per group. Eight-week-old male and female mice (sex-matched between KO and WT group) were used in the ELISA and flow cytometry analysis. Statistical significance was determined using an unpaired two-tailed t-test. Error bars represent the s.d. *p< 0.05, **p < 0.01, ***p<0.001, ****p<0.0001 and ns p>0.05.

Supplementary Fig. 3

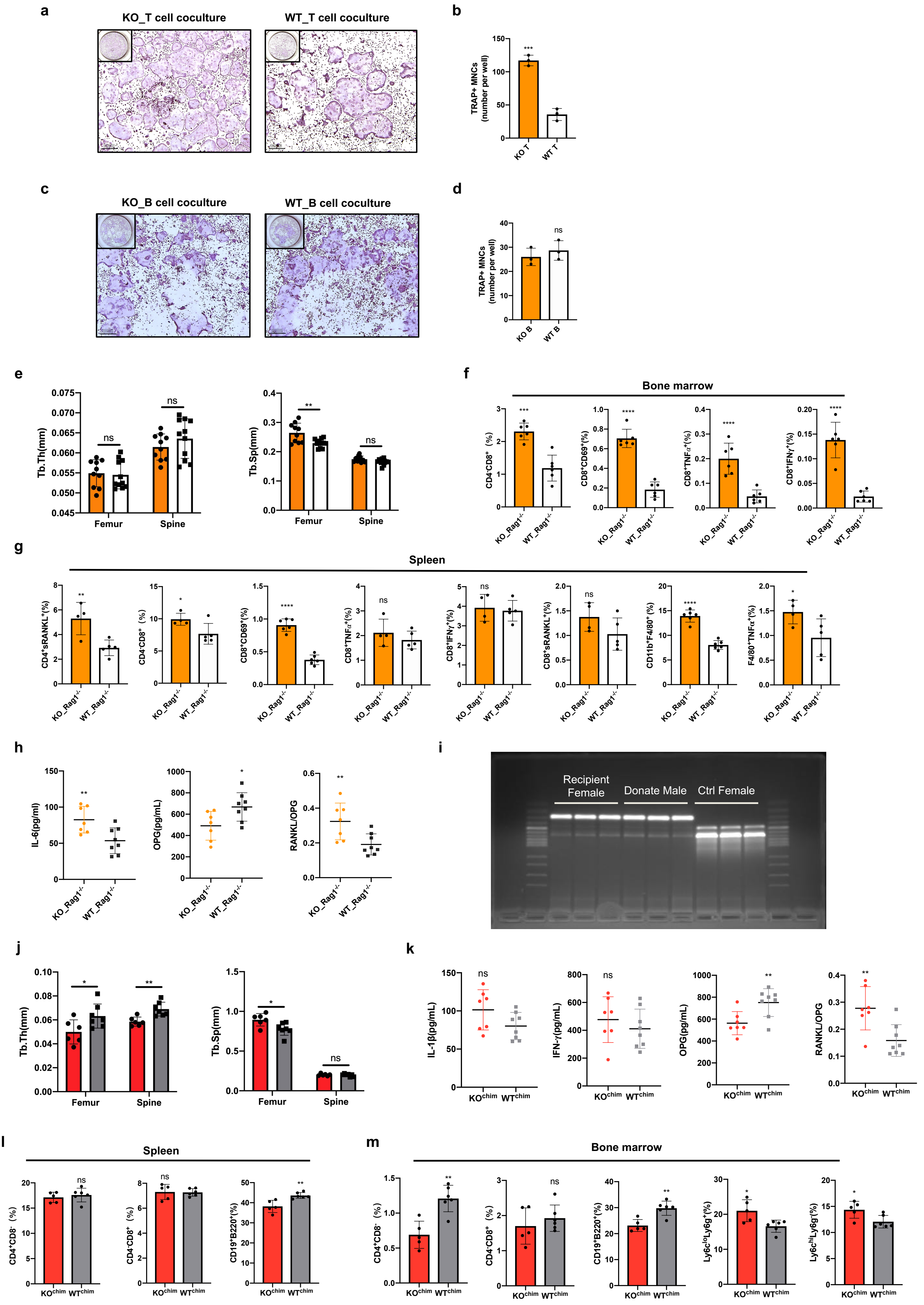

Fig. S3. The systemic inflammation in TLR9-/- mice plays an important role in the osteoclastogenic bone loss.

a-d BMNCs from $Rag1^{-/-}$ mice were cocultured with splenic $CD4^+$ T cells (**a-b**) or B cells (**c-d**) from $TLR9^{-/-}$(KO) and wildtype (WT) mice and stimulated with RANKL and CSF1 in a transwell system. **a** and **c** Representative images of OCLs by TRAP staining at the end of assay. **b** and **d** Quantification of number of $TRAP^+$ multinucleated OCLs. **e-h** Adoptive transfer of $TLR9^{-/-}$ and wildtype splenocytes in $Rag1^{-/-}$ mice. **e** Quantifications of femur and spine Tb.Th and Tb.Sp. n=10 and 11 in KO_$Rag1^{-/-}$ and WT_$Rag1^{-/-}$ group, respectively. **f-g** Proportions of T cell and macrophage populations in the bone marrow (**f**) and spleen (**g**) of KO_$Rag1^{-/-}$ and WT_$Rag1^{-/-}$ mice. n=4-6 mice per group. **h** Circulating levels of IL6, OPG and ratio of RANKL/OPG in KO_$Rag1^{-/-}$ and WT_$Rag1^{-/-}$ group. n=7 and 8 in KO_$Rag1^{-/-}$ and WT_$Rag1^{-/-}$ group, respectively. **i-m** Inflammatory cytokine levels and immune cell phenotypes in bone marrow chimera models. The $TLR9^{-/-}$ and wildtype bone marrow cells was transferred into lethally irradiated wildtype mice to construct the bone marrow chimera models (KO^{chim} and WT^{chim} mice). **i** To verify the cell transfer efficiency in the bone marrow chimera mice, a separate experiment was performed at the same time by transferring bone marrow cells from wildtype male donors to lethally irradiated female wildtype mice. Three weeks after transfer, bone marrow cells from the female recipient mice were harvested and genomic DNA extracted. PCR genotyping of X and Y chromosomes was performed using the bone marrow cell genomic DNA and all the PCR products were amplified using the same set pair of primers with same cycling settings. The gel image showed that the bone marrow cells from female recipient mice had the same genotype as the male donors. Ctrl Female means wildtype female mice with no treatment. One lane represents result from one single mouse. **j** Quantifications of femur and spine Tb.Th and Tb.Sp. n=6 and 7 in KO^{chim} and WT^{chim} group, respectively. **k** Circulating levels of IL1β, IFNγ, OPG and ratio of RANKL/OPG in KO^{chim} and WT^{chim} mice. n=7 and 8 in KO^{chim} and WT^{chim} group, respectively. **l-m** Flow cytometry analysis of immune cell populations in the spleen (**l**) and bone marrow (**m**) of KO^{chim} and WT^{chim} mice. In all panels, n=5 and 6 in KO^{chim} and WT^{chim} group, respectively. The numbers represent the frequencies in total splenocytes or bone marrow cells. Statistical significance was determined using an unpaired two-tailed t-test. Error bars represent the s.d. *$p< 0.05$, **$p < 0.01$, ***$p<0.001$, ****$p<0.0001$ and ns $p>0.05$.

Supplementary Fig. 4

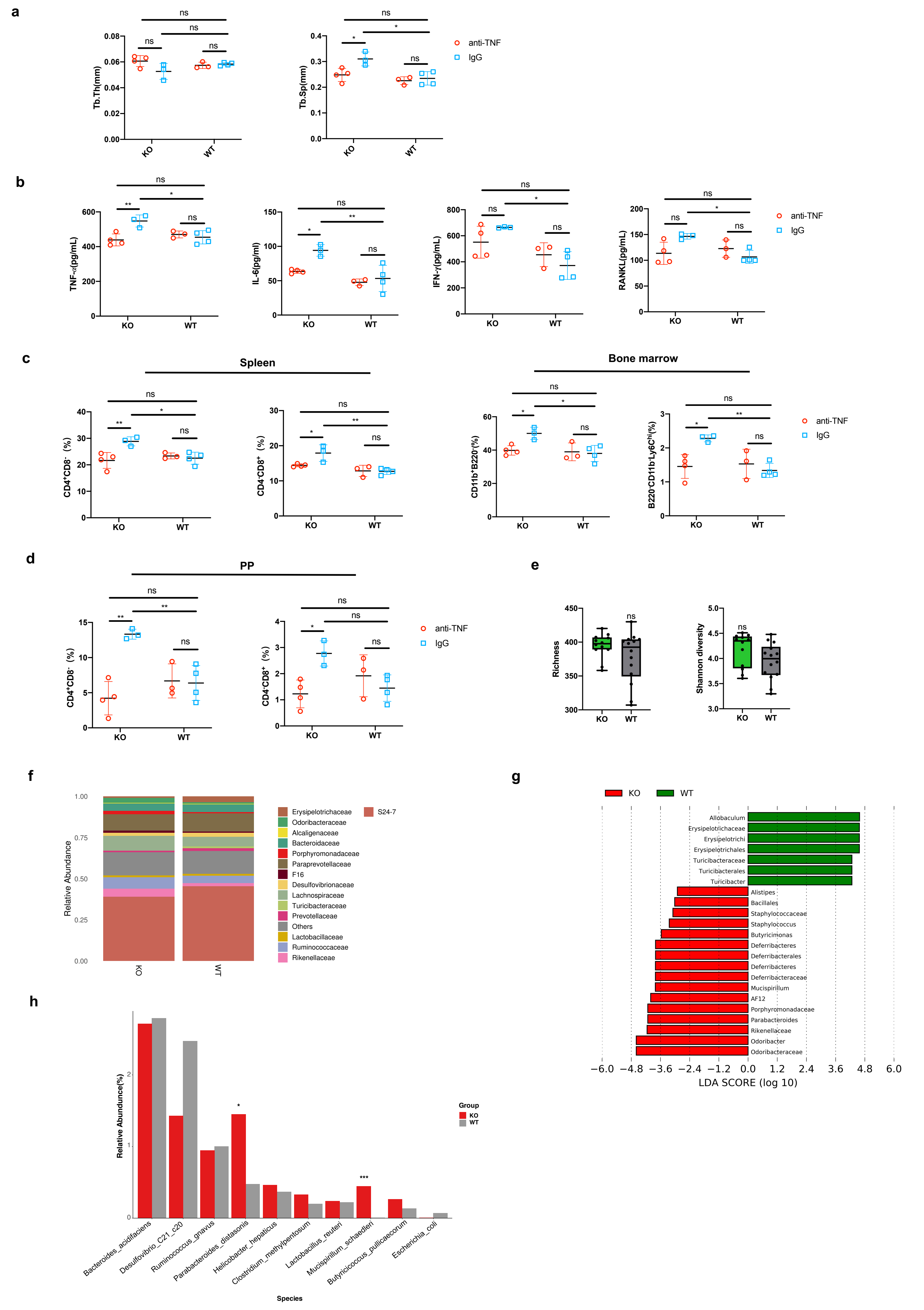

Fig. S4. Anti-TNFα therapy in TLR9$^{-/-}$ mice and the altered gut microbiota composition in TLR9$^{-/-}$ mice. The anti-TNFα therapy experiment. In all panels, n=3 in TNFα_WT and IgG_KO group; n=4 in TNFα_KO and IgG_WT group. **a** Quantifications of femur Tb.Th and Tb.Sp. **b** Circulating levels of IL6, TNFα, IFNγ and RANKL in each group. **c** Proportions of splenic T cells and bone marrow myeloid cells analyzed by flow cytometry in each group. The numbers represent the frequencies in total splenocytes or bone marrow cells. **d** Proportions of T cells in PP analyzed by flow cytometry. The numbers represent the frequencies in total PP cells. **e-h** Deficiency of TLR9 altered gut microbiota composition. **e** Estimated richness (left panel) and Shannon diversity (right panel) of microbiota samples as in Fig. 3d. **f** Bar chart of the relative microbiome abundance at family level from samples as in Fig. 3d. **g** LDA scores of LEfSe analysis in KO and WT group. **h** Top ten abundant species were analyzed and differential abundance were compared between KO and WT mice. For determination of statistical significance, two-way ANOVA with multiple comparisons (Turkey's test) was used in **a-d**. Mann-Whitney t test was used in **e** and **h**. An unpaired two-tailed t-test was applied in other panels. Error bars represent the s.d. *$p < 0.05$, **$p < 0.01$, ***$p < 0.001$ and ns $p > 0.05$.

Supplementary Fig. 5

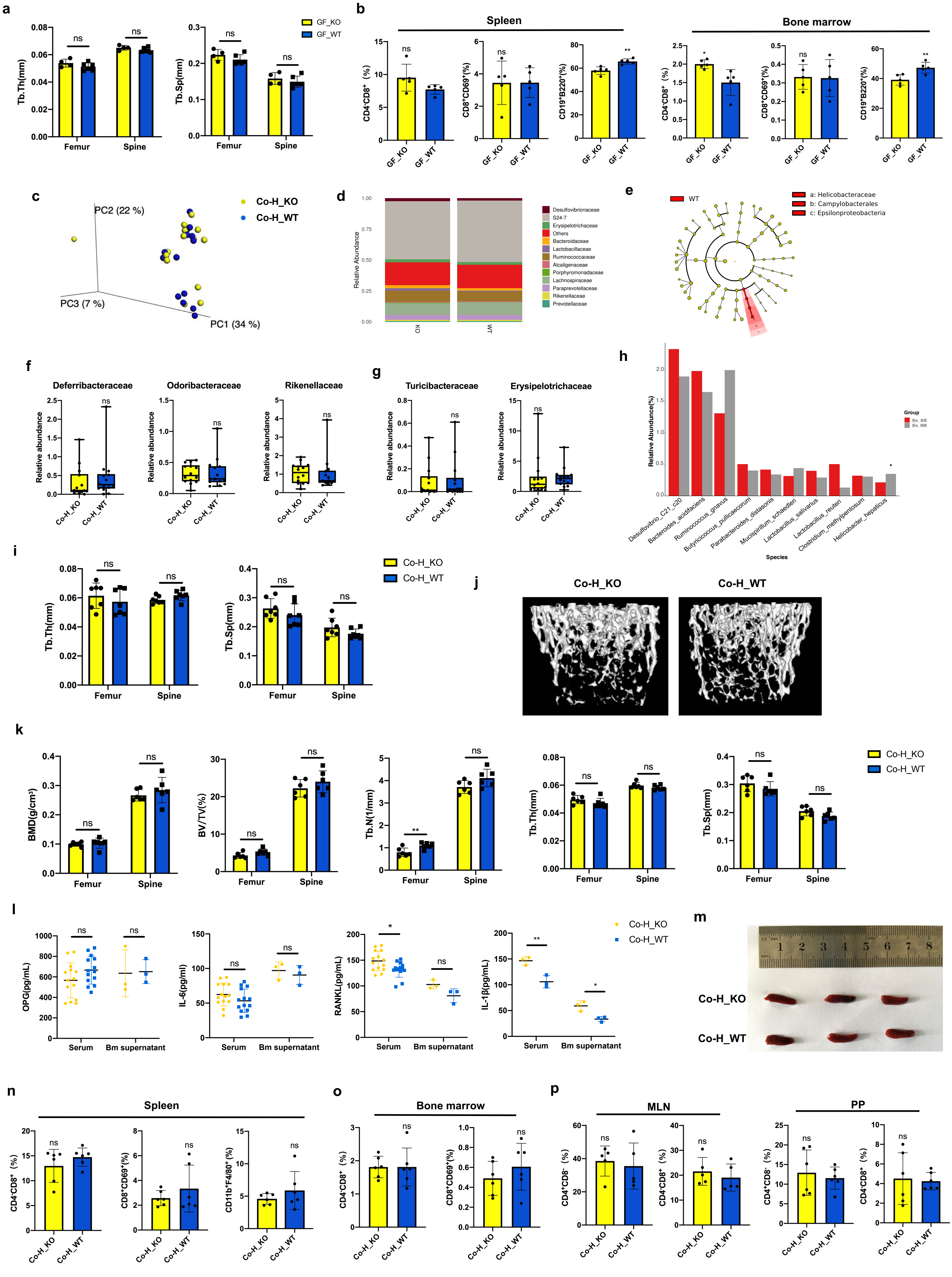

Fig. S5. Gut microbiota played an important role in the systemic inflammation and bone loss in TLR9$^{-/-}$ mice.

a Quantifications of femur and spine Tb.Th and Tb.Sp in the 8-week-old male GF mice. n=4 and 6 in the GF TLR9$^{-/-}$ and wildtype group, respectively. **b** Proportions of the CD8^{+} T cells and B cells in the spleen and bone marrow of germ-free (GF) TLR9$^{-/-}$ and wildtype mice. n=5 per group. **c-o** The cohousing experiment. **c** 3-Dimentional PCoA of 16S rRNA sequencing of fecal microbiota from cohoused TLR9$^{-/-}$(Co-H_KO) and wildtype (Co-H_WT) mice. n=14 per group. Each dot represents a fecal microbiota from one mouse. **d** Bar chart of the relative microbiome abundance at family level from mice as in **c**. **e** LEfSe analysis shows bacteria that were differentially abundant in Co-H_KO and Co-H_WT group. **f-g** Relative abundance of the families which showed significant differences in Fig. 3e, f. Staphylococcaceae was not detected in feces samples after cohousing. Boxplots represent median and quantiles. n=14 per group in all panels. **h** Top ten abundant species in Co-H_KO and Co-H_WT mice. **i** Quantifications of femur and spine Tb.Th and Tb.Sp in the male Co-H_KO and Co-H_WT mice. n=7 per group in all panels. **j-k** Trabecular bone microarchitecture of femurs and L3 vertebrae of cohoused female TLR9$^{-/-}$(Co-H_KO) and wildtype (Co-H_WT) mice. **j** Representative 3-dimensional μCT reconstructions of examined femurs from each group. **k** Quantifications of femur and spine BMD, BV/TV, Tb. N, Tb.Th and Tb.Sp. n=6 per group in all panels. **l** Levels of OPG, IL6, RANKL and IL1β in serum and bone marrow supernatants from Co-H_KO and Co-H_WT mice. Serum, n=14 per group for OPG, IL6 and IFNγ; n=3 per group for IL1β; Bone marrow supernatant, n=3 per group in all panels. **m** Representative image shows the sizes of spleens from 12-week-old male Co-H_KO and Co-H_WT mice. **n** Flow cytometry analysis of T cell and myeloid cell populations in the spleen of Co-H_KO and Co-H_WT mice. n=6 per group in all panels. **o** Flow cytometry analysis of bone marrow CD8^{+} T cells in Co-H_KO and Co-H_WT mice. n=5-6 per group in all panels. **p** T cell populations in MLN and PP by flow cytometry. MLN, n=5 per group; PP, n=6 per group. In **n-p**, the numbers represent the frequencies in total splenocytes, bone marrow cells, MLN or PP cells. Twelve-week-old male and female mice (sex-matched between the groups) were used in the gut microbiome, ELISA and flow cytometry analysis in **c-h**, **l** and **n-p**. Mann-Whitney t test was used in **f-h**. Unpaired two-tailed t-test was used in other panels. Error bars represent the s.d. *$p< 0.05$, **$p < 0.01$ and ns $p>0.05$.

Supplementary Fig. 6

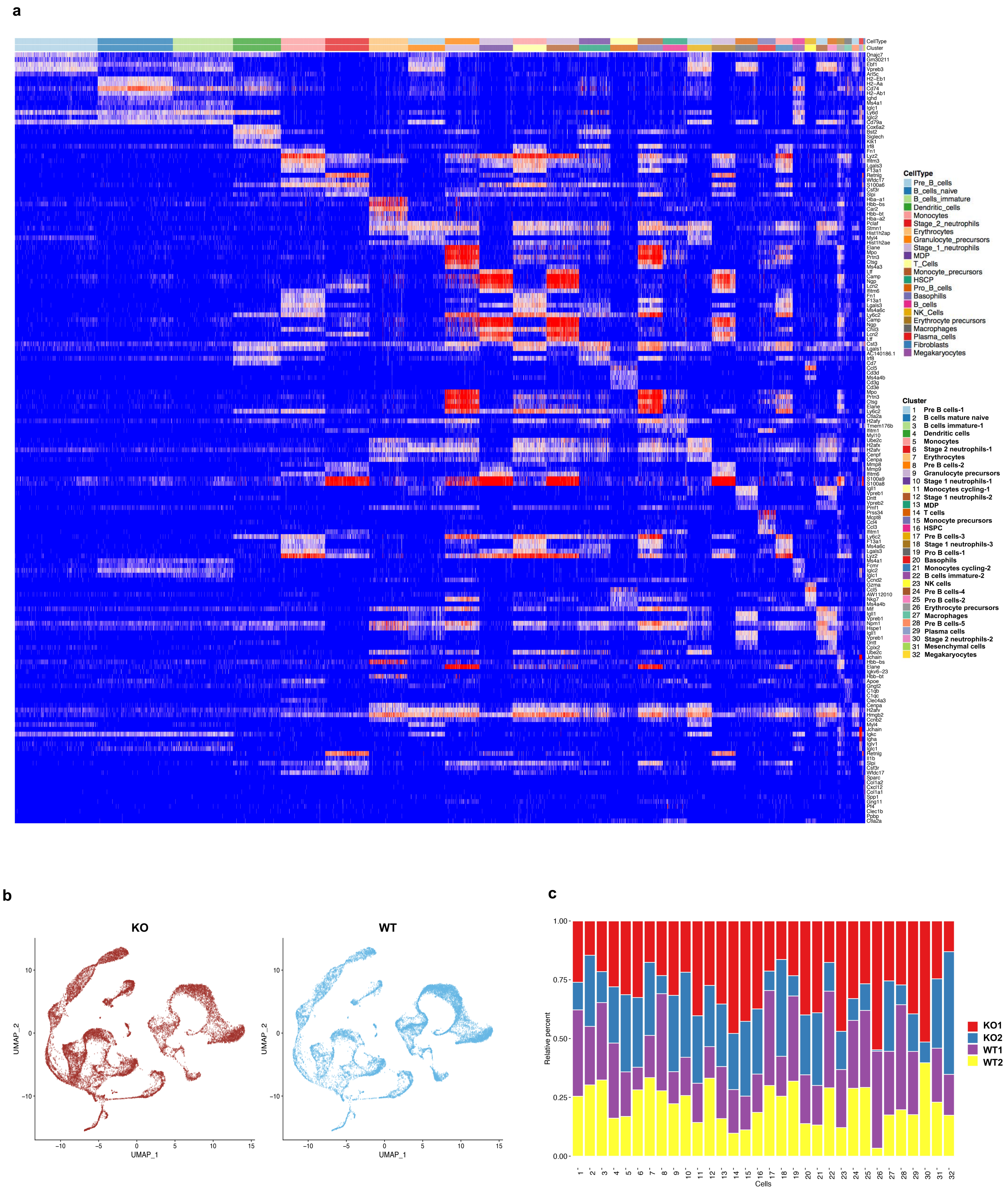

Fig. S6. Clustering of cells and cell markers in scRNA-seq.

a The cell-type markers. The relative expression level of genes across cells is shown, sorted by cell type and cluster. Marker genes were identified in an unbiased fashion (Wilcoxon rank-sum test, $p < 0.05$, and fold change >1.5) and only the top five are shown in the figure. **b** UMAP clustering as in Fig. 5a, but colored by group (KO and WT). **c** Cell distribution analysis colored by replicates in each cluster. X-axis represents the cluster number.

Supplementary Fig. 7

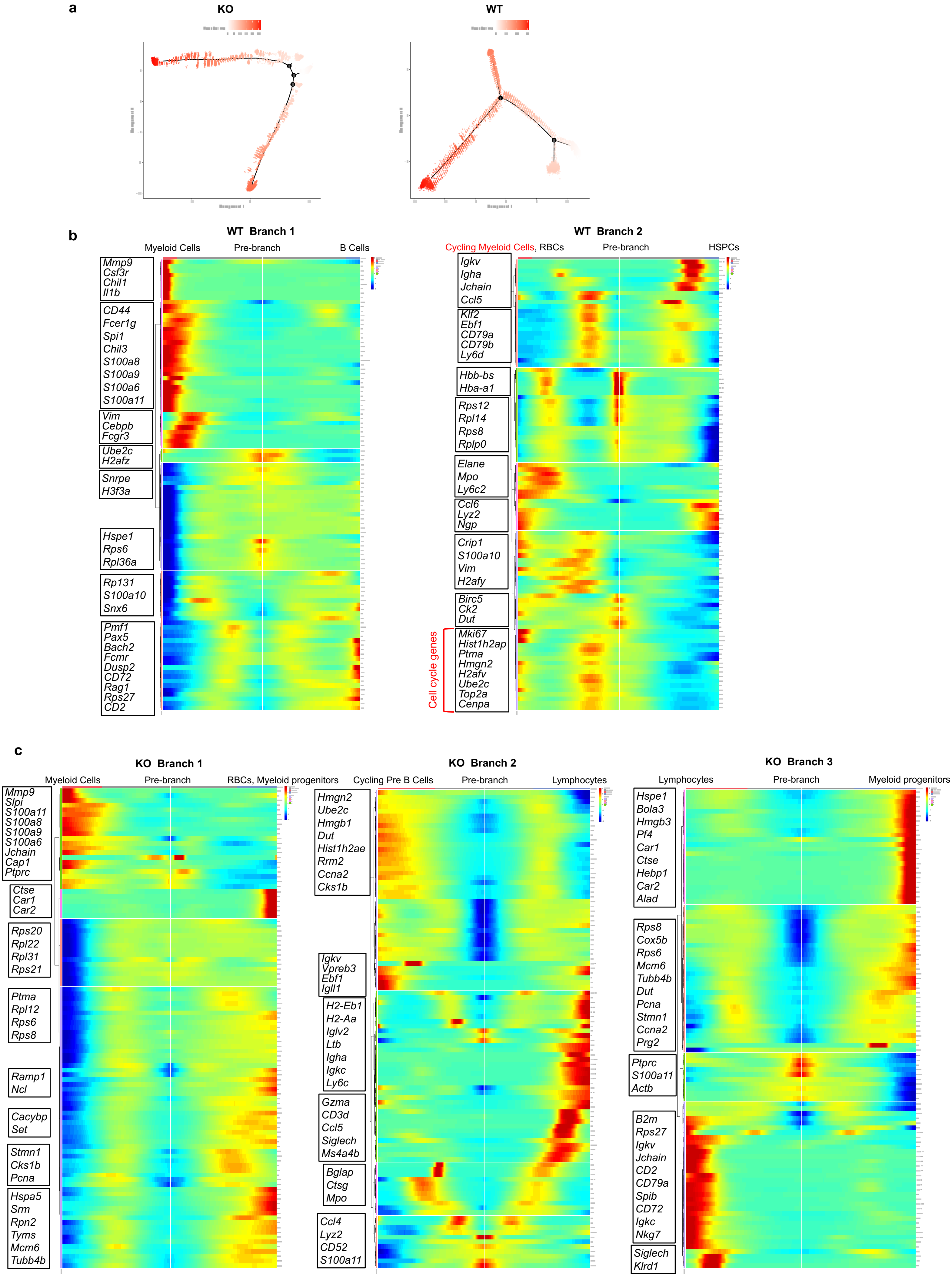

Fig. S7. Trajectory analysis of bone marrow cells' development in TLR9$^{-/-}$ and wildtype mice

a Trajectory analysis of TLR9$^{-/-}$(KO) and wildtype (WT) cells colored by pseudotime. **b-c** Heatmaps of the significantly changed genes discovered by the Branch expression analysis modeling (BEAM) function from monocle in each branch point of KO and WT cells. The red color in **b** marks the cell cycle genes expressed by cycling myeloid cells in the branch 2 of WT cells.

Supplementary Fig. 8

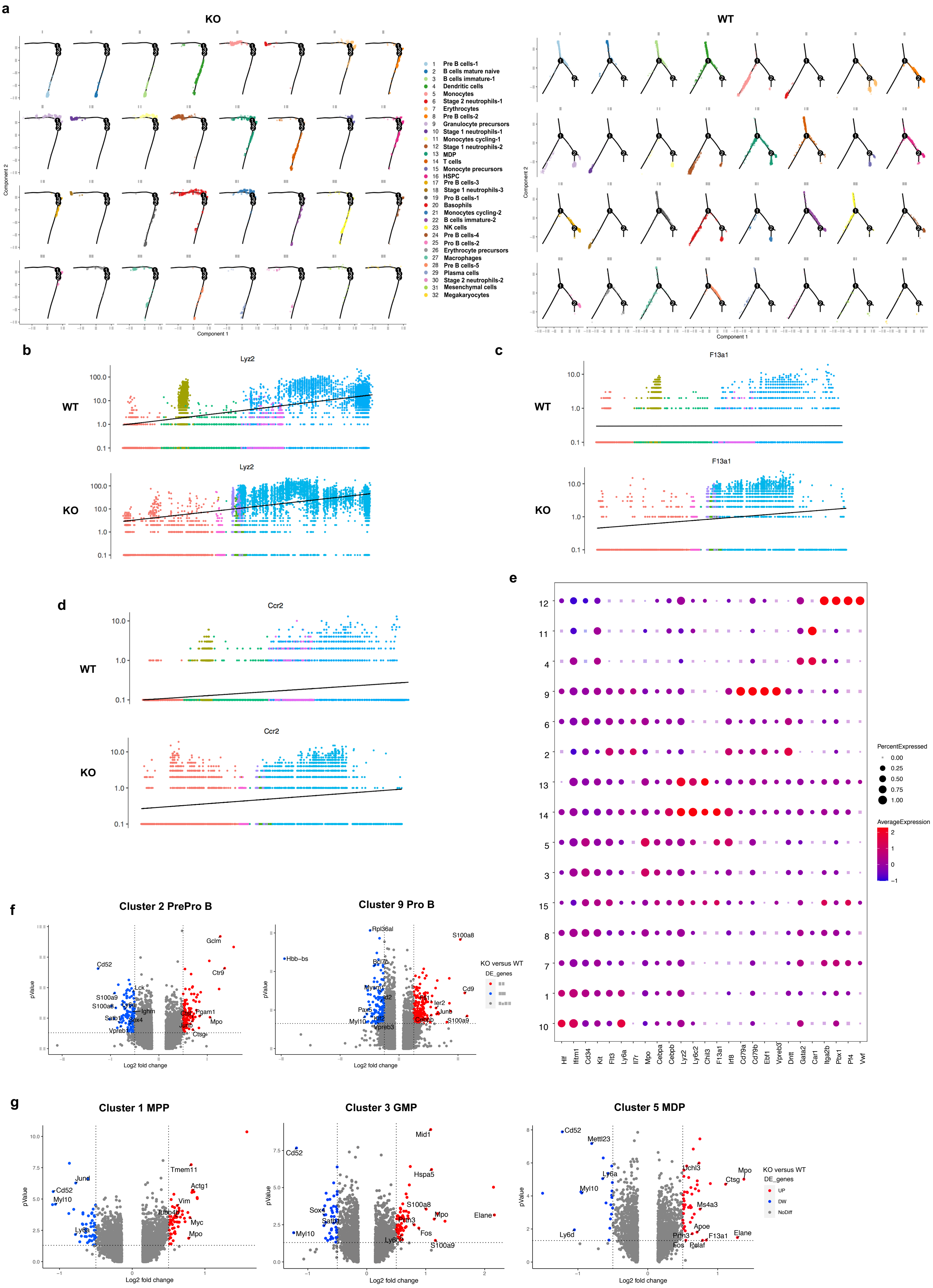

Fig. S8. scRNA-seq analysis of bone marrow HSPCs

a Trajectory analysis of TLR9$^{-/-}$ (KO, left panel) and wildtype (WT, right panel) cells shown by clusters. **b-d** Expression of monocyte marker genes Lyz2 (**b**), F13a1 (**c**) and Ccr2 (**d**) across the pseudotime (from left to right) in WT and KO bone marrow cells. Each dot represents one cell. **e** Dot plot shows marker gene expression in the subclusters of HSPCs. **f-g** Volcano plots show the DEGs between KO and WT cells in clusters of PrePro B cells (cluster 2), Pro B cells (Cluster 9), MPP (cluster 1), GMP (cluster 3) and MDP (cluster 5). DEGs with Log_2[fold change]>0.5 and p<0.05 were considered significant. The cluster numbers and names correspond to those of HSPC subclusters in Fig. 5i. Red dots, upregulated genes in TLR9$^{-/-}$ cells; Blue dots, downregulated genes in TLR9$^{-/-}$ cells.

Supplementary Fig. 9

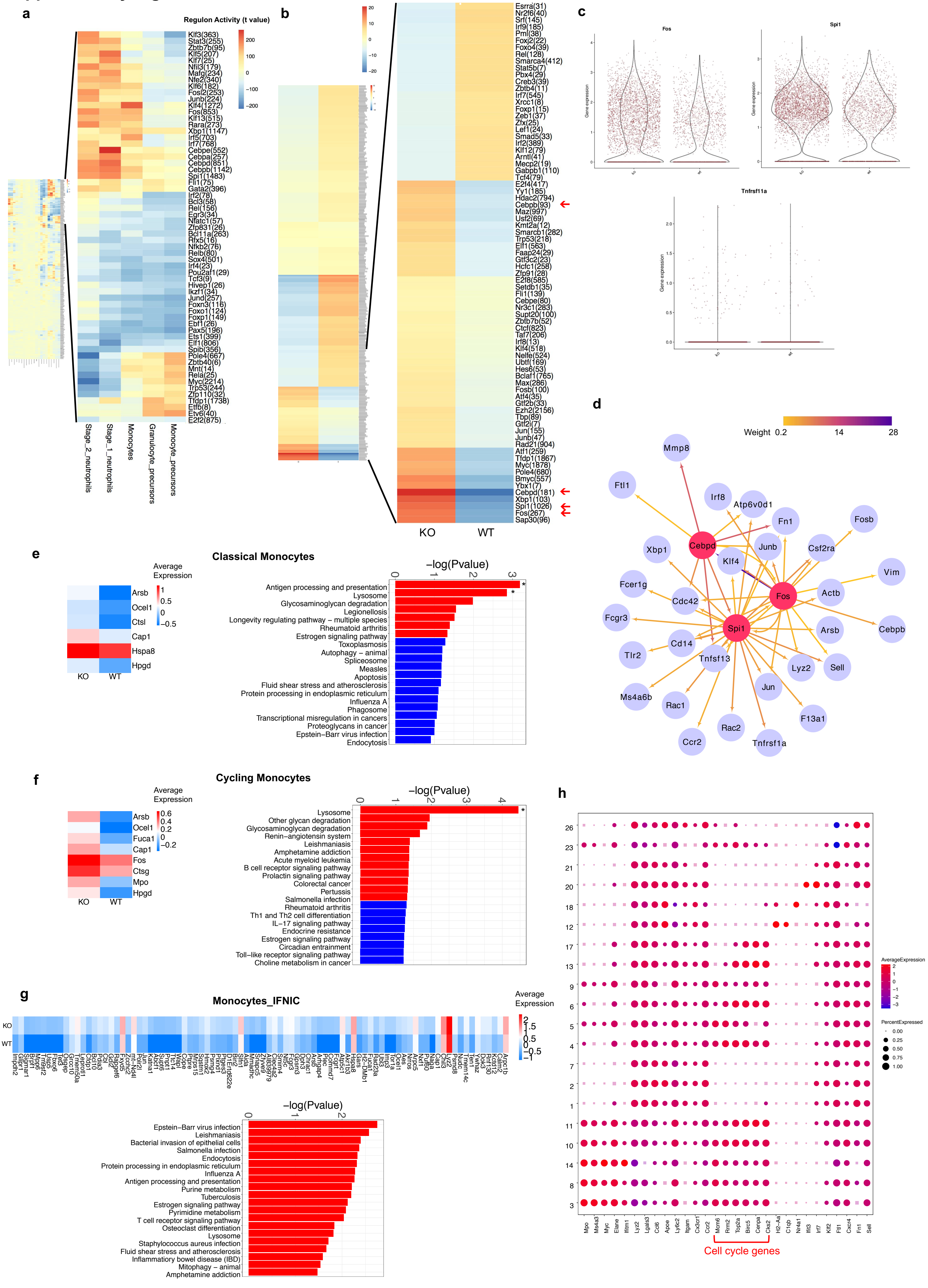

Fig. S9. scRNA-seq analysis of bone marrow monocyte/macrophages.

a SCENIC analysis shows activities of signature transcription factors (TFs) in the indicated cell types (X axis). Clusters with the same cell type in Fig. 5a were pooled together for analysis. **b** and **c** Clusters of monocyte/macrophages (cluster 5,11,15,17,21 in Fig. 5a) were pooled together for analysis. **b** SCENIC analysis shows differences of TFs' activities between TLR9$^{-/-}$(KO) and wildtype (WT) monocyte/macrophages. **c** Expression of Fos, Spi1 and RANK (Tnfrsf11a) in KO and WT monocyte/macrophages. Each dot represents one cell. **d** Transcriptional network of Fos, Cebpd and Spi1 in monocyte/macrophages based on SCENIC analysis. Selected monocyte-related target genes were shown in light purple color. The arrow colors represent the weight of each TF-target pair. **e-g** Significantly-upregulated genes (Fold Change>1.5 and p<0.05) in KO monocytes and corresponding functional enrichment according to KEGG pathways. For the KEGG enrichments, red bars, p<0.05; blue Bars, p>0.05; *, FDR<0.05 (Bonferroni correction). **e** Classical monocytes (pool of cluster 1, 2 and 7 in Fig. 7a). **f** Cycling monocytes (pool of cluster 4, 5, 6, 9, 13 and 17 in Fig. 7a). **g** Monocytes_IFNIC (cluster 20 in Fig. 7a). **h** Dot plot shows marker gene expression in the subclusters of bone marrow monocyte/macrophages.

Supplementary Fig. 10

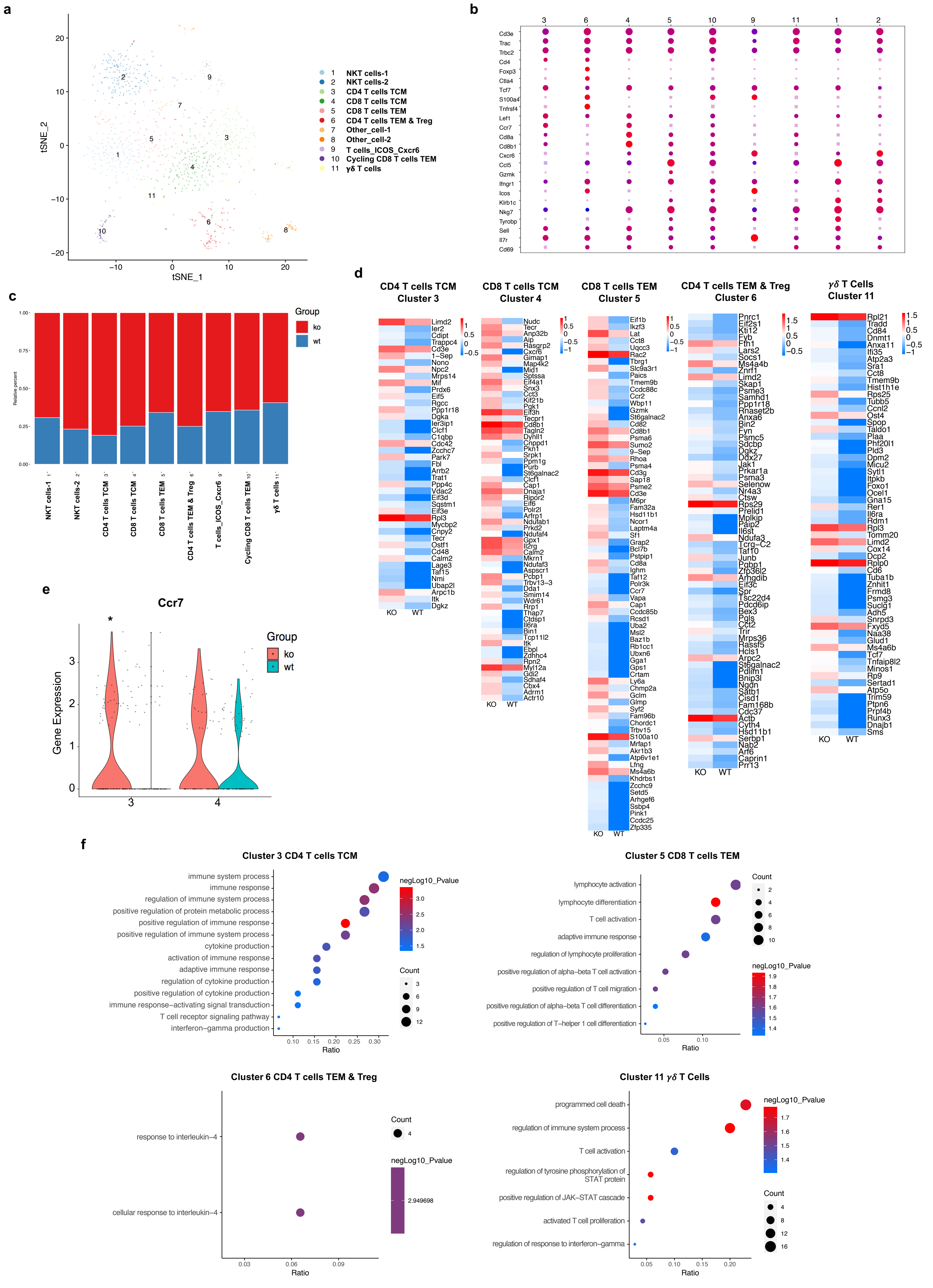

Fig. S10. scRNA-seq analysis of bone marrow T cells.

a The reclustered T cells (cluster 14 in Fig. 5a). **b** Dot plot shows marker gene expression in the subclusters of bone marrow T cells. **c** Proportion of $TLR9^{-/-}$(KO) and wildtype (WT) cells in T cell subclusters as in **a**. (The non-T cell clusters were excluded from the analysis). X axis represents cluster number and cell type. **d** Heatmaps showing KO-upregulated genes with significance (Fold Change>1.5 and p<0.05) in the indicated cell types. **e** Expression of Ccr7 in subcluster 3 (CD4 TCM) and 4 (CD8 TCM). **f** Dot plots showing Gene Ontology (GO) functional enrichment of genes in **c**. (only selected biological function terms with FDR< 0.05 [Bonferroni correction] are shown). Cluster 4 had no functional enrichment terms with FDR< 0.05. The population nomenclature and cluster numbers correspond to those in **a**. Wilcoxon test was used to determine the significance in **e**. *p< 0.05.

Supplementary Fig. 11

a

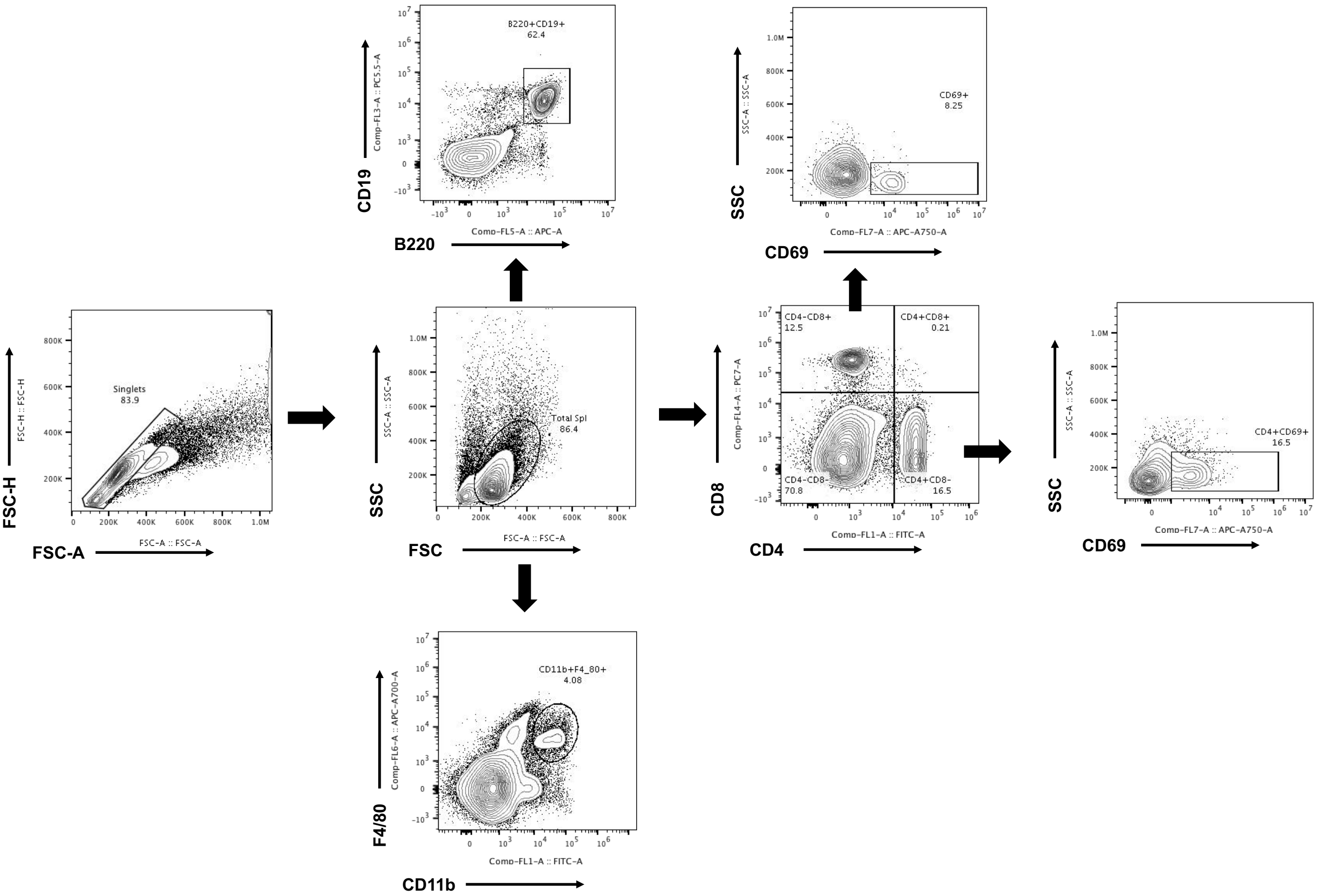

b

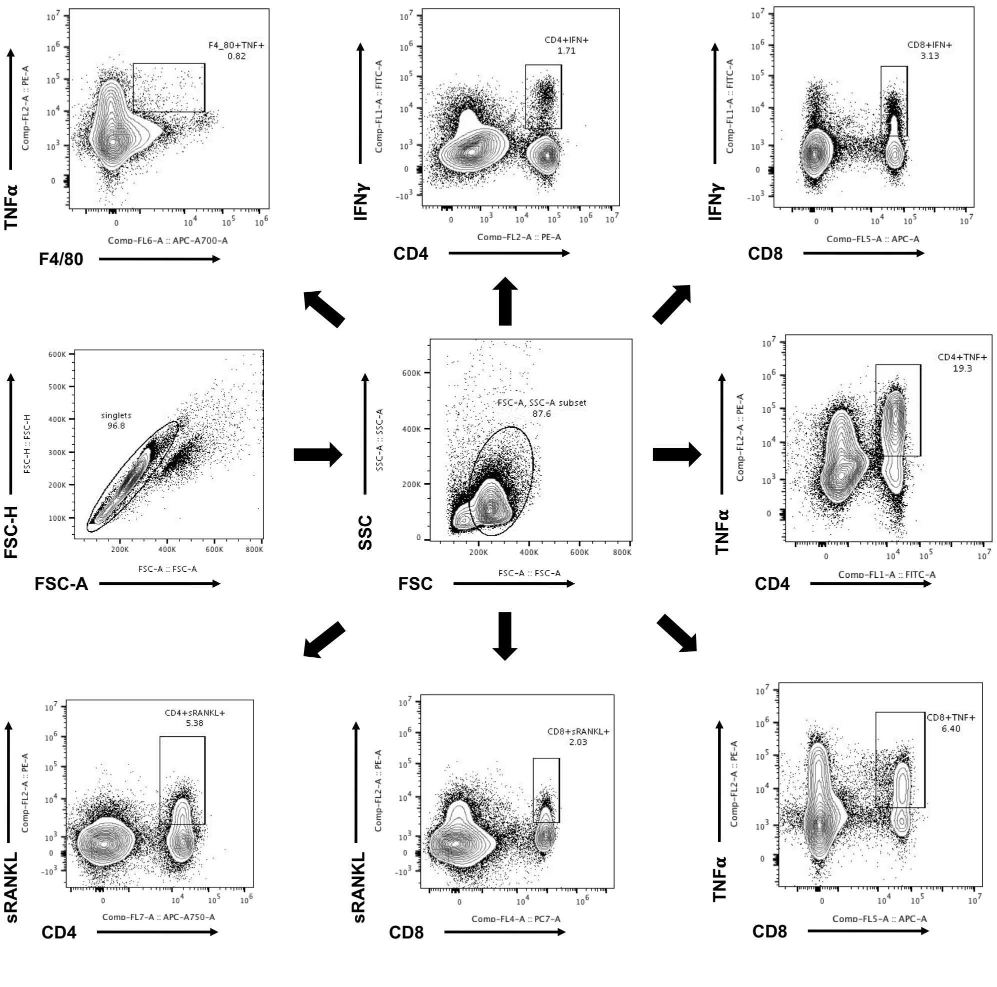

Fig. S11. Flow cytometry gating strategies for analysis of splenocytes.

a The contour plots show the gating strategies for surface marker analysis. **b** The contour plots show the gating strategies for intracellular cytokine analysis.

Supplementary Fig. 12

a

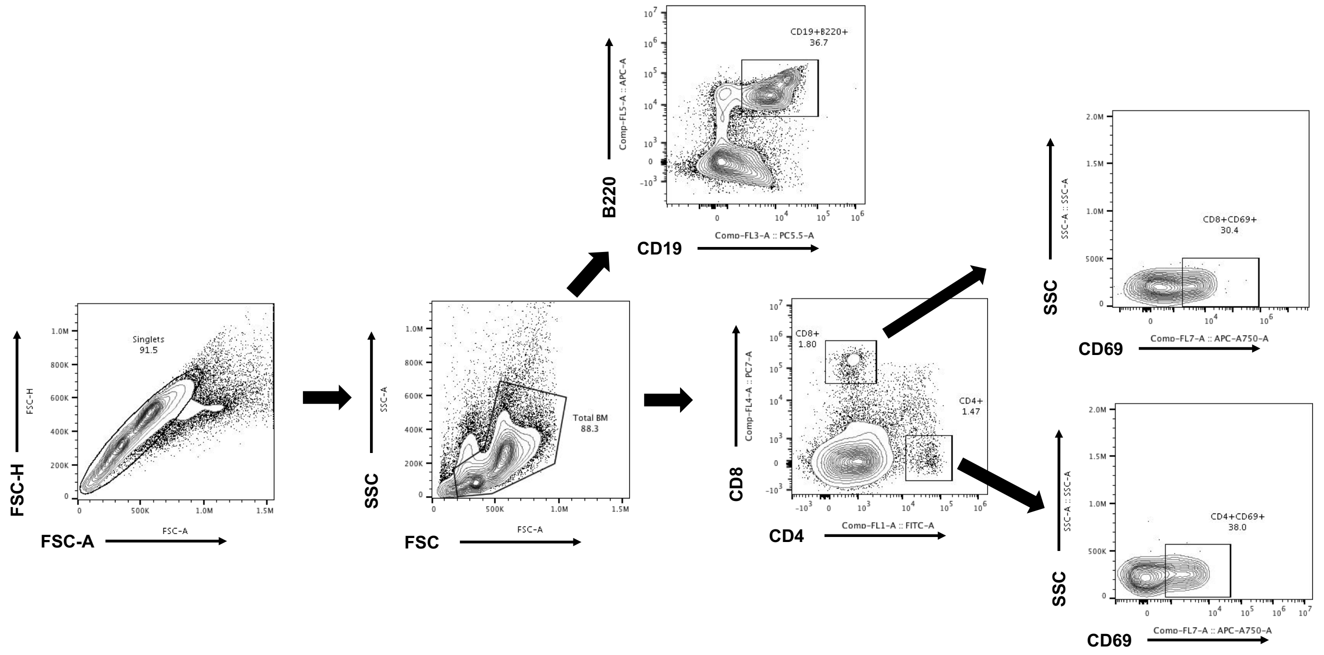

b

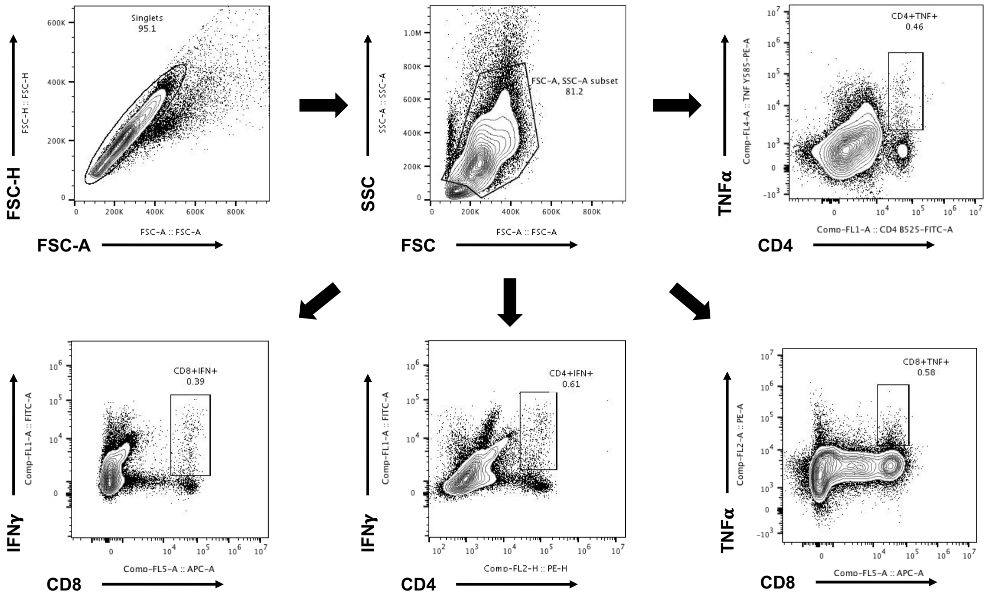

c

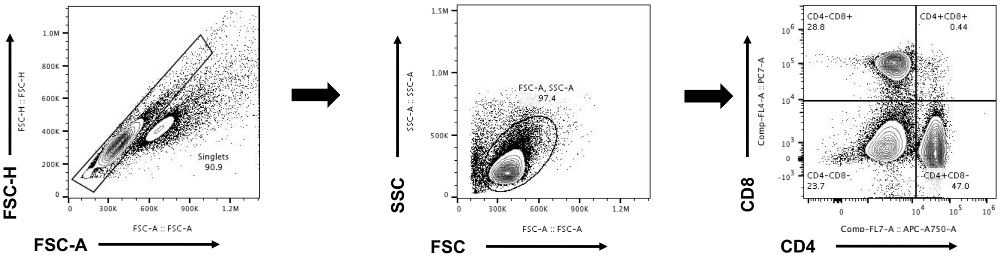

d

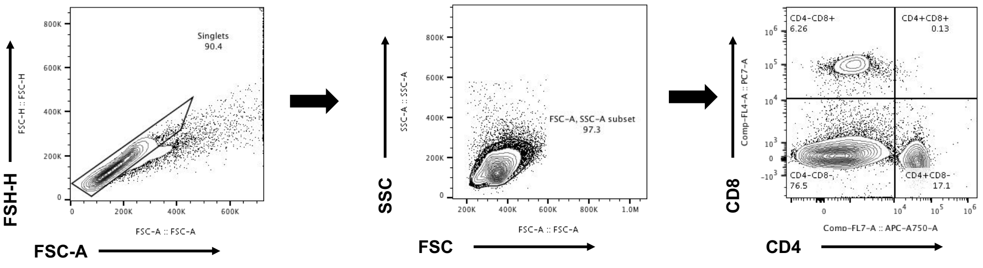

Fig. S12. Flow cytometry gating strategies for analysis of bone marrow, MLN and PP cells.

a The contour plots show the gating strategies for surface marker analysis in bone marrow cells. **b** The contour plots show the gating strategies for intracellular cytokine analysis in bone marrow cells. **c** The contour plots show the gating strategies for MLN cells. **d** The contour plots show the gating strategies for PP cells.

Supplementary Fig. 13

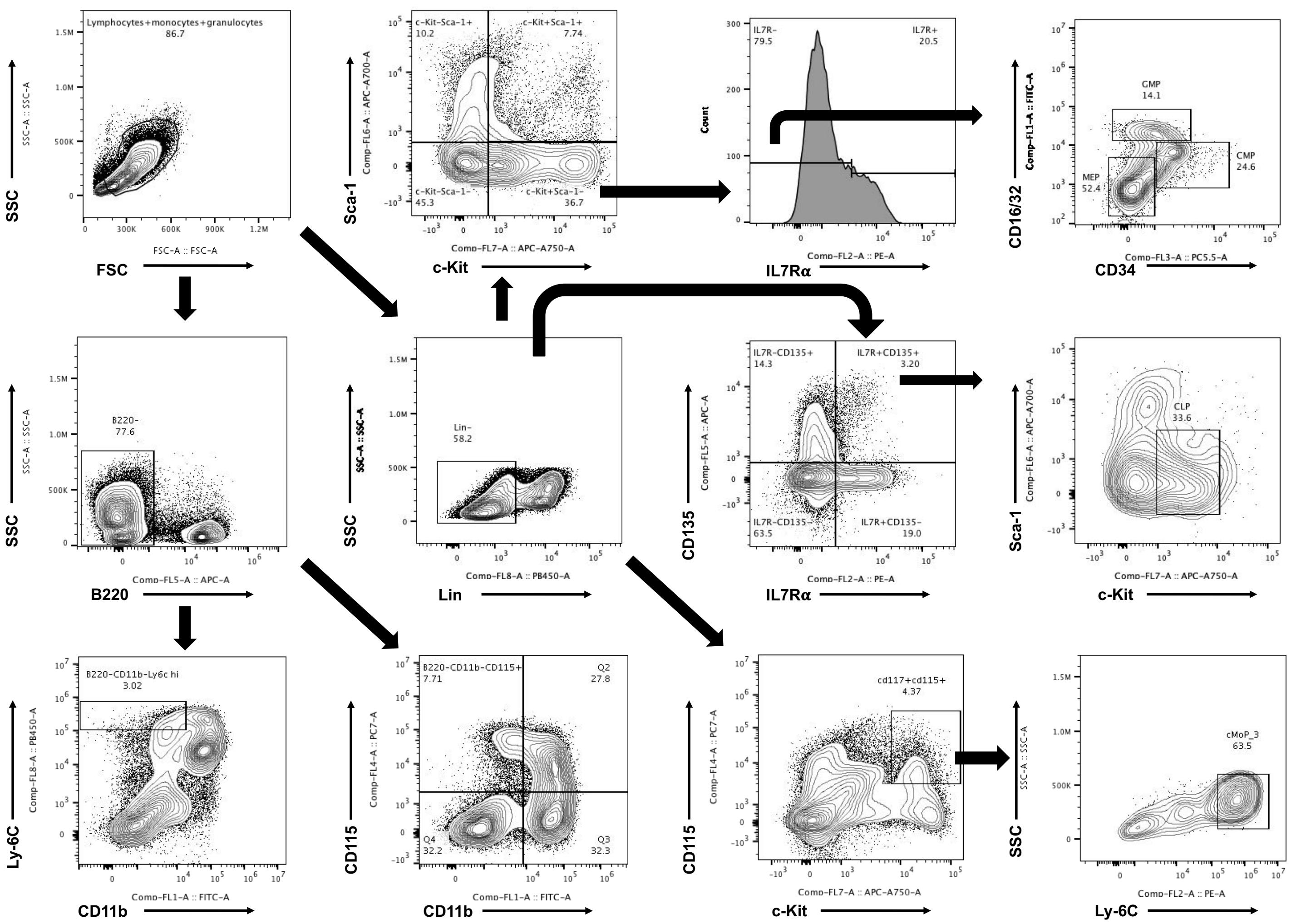

Fig. S13. Flow cytometry gating strategies for analysis of bone marrow HSPCs and osteoclast progenitor cells.

Table S1.

List of primers used for real-time PCR.

Gene	Forward primer	Reverse primer
GAPDH	ACCCAGAAGACTGTGGATGG	ACCCAGAAGACTGTGGATGG
CTSK	CCAGTGGGAGCTATGGAAGA	AAGTGGTTCATGGCCAGTTC
RANK	TAGGACGTCAGGCCAAAGGACAAA	AGGGCCTACTGCCTAAGTGTGTTT
NFATc1	AGATGGTGCTGTCTGGCCATAACT	TGGTTGCGGAAAGGTGGTATCTCA
ALP	TCAGGGCAATGAGGTCACAT	CCTCTGGTGGCATCTCGTTA
COL1A1	ATAAGTCCCTTCCTGCCCAC	TGGGACATTTCAGCATTGCC
RUNX-2	GCCCAGGCGTATTTCAGATG	GGTAAAGGTGGCTGGGTAGT
OCN	CCCTGAGTCTGACAAAGCCT	GCGGTCTTCAAGCCATACTG
OSX	TCGGGGAAGAAGAAGCCAAT	CAATAGGAGAGAGCGAGGGG
TLR9	CCTGAAGTCTGTACCCCGTT	TCTGGGCTCAATGGTCATGT
